# Fatty acid acylation regulates trafficking of the unusual *Plasmodium falciparum* calpain to the nucleolus

**DOI:** 10.1111/j.1365-2958.2009.06639.x

**Published:** 2009-03-02

**Authors:** Ilaria Russo, Anna Oksman, Daniel E Goldberg

**Affiliations:** Howard Hughes Medical Institute, Washington University School of Medicine, Departments of Molecular Microbiology and MedicineSt Louis, Missouri 63110, USA

## Abstract

The *Plasmodium falciparum* genome encodes a single calpain. By generating *P. falciparum* clones expressing C-terminally tagged calpain, we localized this protein to the nucleolus. Pf_calpain possesses an unusual and long N-terminal domain in which we identified three subregions that are highly conserved among *Plasmodium* species. Two have putative targeting signals: a myristoylation motif and a nuclear localization sequence. We assessed their functionality. Our data show that the nuclear localization sequence is an active nuclear import motif that contains an embedded signal conferring nucleolar localization on various chimeras. The N-terminus is myristoylated at Gly2 and palmitoylated at Cys3 and Cys22. Palmitoylation status has an important role in dictating *P. falciparum* calpain localization. The targeting signals function in mammalian cells as well as in the parasite. *P. falciparum* calpain is a unique nucleolar protein with an interesting mechanism of targeting.

## Introduction

Cysteine proteases that belong to the calpain superfamily possess a well-conserved catalytic domain (Sorimachi, 2004). However, these enzymes otherwise exhibit vast divergence of sequence and domain structure (Croall and Ersfeld, 2007). Calpains can be divided into typical (such as human calpains 1 and 2) and atypical (lacking a calcium-binding domain IV). They are found in all prokaryotic and eukaryotic kingdoms. Some species, such as humans, have a dozen or more calpains.

Calpains have been implicated in a variety of cellular processes such as muscle function, cell signalling, migration, attachment, cell death, transformation, cell-cycle regulation, differentiation, stress responses, development (Beckerle *et al*., 1987; Sorimachi *et al*., 1997; 2004; Carragher and Frame, 2002; Kulkarni *et al*., 2002; Janossy *et al*., 2004). However, the precise physiological role of most of the calpains in these processes is poorly understood (Perrin and Huttenlocher, 2002; Croall and Ersfeld, 2007). Different calpains are found in varied locations: cytoplasm, nucleus, mitochondrion, cytoskeleton- and membrane-associated (Emori and Saigo, 1994; Arrington *et al*., 2006; Fernandez-Montalvan *et al*., 2006; Shao *et al*., 2006). *Plasmodium falciparum* possesses a single unusual calpain, Pf_calpain. Its gene is essential and appears to play a role in pre-S phase development (Russo *et al*., 2009). The protein has a very large N-terminus that is only found in apicomplexan calpains and those of some other alveolates (Russo *et al*., 2009). In comparing N-terminal sequences between different species, we have found three regions of high homology. Two of these are important for controlling Pf_calpain location in the cell, and serve to effect targeting of this protein to the nucleolus in *Plasmodium* as well as in mammalian cells. This location may be important for Pf_calpain function in cell cycle progression.

## Results

### C-terminal tagging of Pf_calpain

**Fig. 1:**
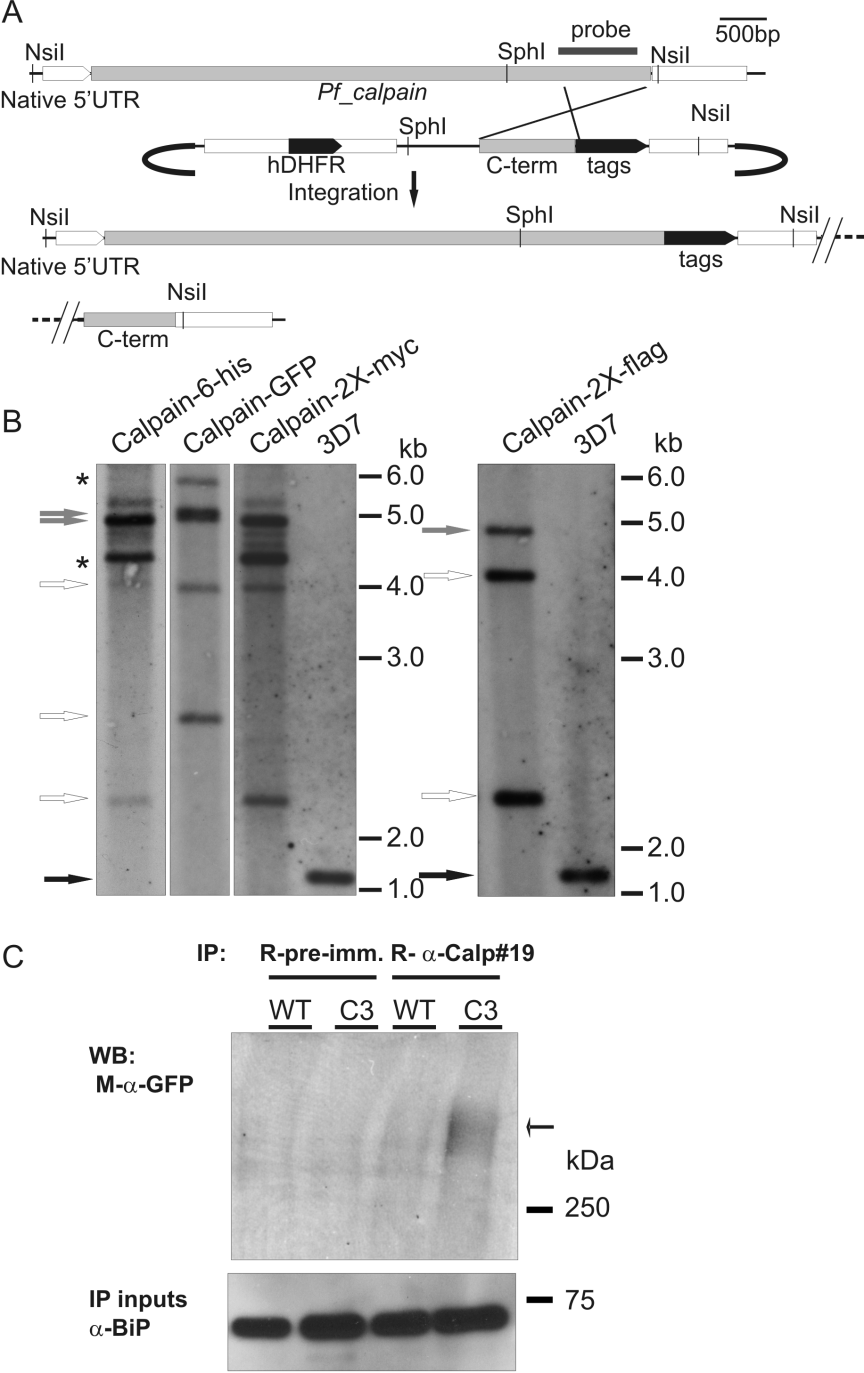
Analysis of Pf_calpain expression using C-terminally tagged chimeras. A. Strategy used to create C-terminally tagged calpains by integration at the 3′ end of the endogenous locus. We generated a set of plasmids containing 1.3 kb of sequence from the 3′ end of the Pf_calpain ORF. Downstream of this sequence we placed different tags in frame: 6-his, GFP, 2×-myc and 2×-flag. A human dihydrofolate reductase (hDHFR) cassette was used for positive drug selection. The relative positions of NsiI and SphI restriction sites, used to detect integration, as well as of the probe are indicated. B. Southern blots of restricted genomic DNA. Calpain-6-his, calpain-GFP, calpain-2×-myc and calpain-2×-flag integrant clones gave the bands expected of a single-crossover recombination (white arrows) and plasmid (grey arrows). The asterisks indicate extra bands probably due to concatamerization with plasmid rearrangement, a frequent event in *P. falciparum*. The 3D7 parental strain shows a single band (black arrows) of the correct size, which is absent in the clones. The difference in size of the smallest DNA fragment reflects the difference in size between GFP and the smaller tags. C. Immunoprecipitated calpain was detected by Western blot analysis. We pulled down calpain using anti-calpain antiserum #19, including as control its pre-immune serum (right panel). For each immunoprecipitation, we used 5 × 10^8^ asynchronous parasites of the parental strain 3D7 (WT) or the calpain-GFP clone (C3). BiP content of each lysate is shown in the panel below. Calpain-GFP was detected by the use of a third antibody, mouse anti-GFP. Pf_calpain is an extremely low-abundance protein that has an apparent molecular weight over 250 kDa (black arrow).

The *P. falciparum* genome contains a single putative calpain gene at the locus MAL13P1.310 (Wu *et al*., 2003; Croall and Ersfeld, 2007). The sequencing of its cDNA reveals an uninterrupted ORF that encodes a 242 kDa predicted protein (GenBank 432832). To study the enzyme *in vivo* while altering its physiological regulation as little as possible, we tagged the Pf_calpain C-terminus by homologous single-crossover recombination at the endogenous locus. We constructed a set of vectors containing a hDHFR drug-resistance cassette and 1.3 kb of the Pf_calpain coding region 3′ end. Downstream to this we introduced, in frame, sequences encoding GFP, 6-his, 2×-myc or 2×-flag (Fig. 1A). After two cycles on and off drug, we obtained successful integration of all constructs (Fig. 1B).

To visualize expression of Pf_calpain required immunoprecipitation of the protein from a large number of parasites (5 × 10^8^) followed by Western blot (WB) detection (Fig. 1C). Mouse anti-GFP antibody recognized a single band after immunoprecipitation with a rabbit anti-calpain antiserum that is specific for a N-terminal peptide (Fig. S1). Similar electrophoretic mobility was observed for the same clone immunoprecipitated with rabbit anti-GFP antibodies and for the his-tagged calpain clone (data not shown). Pf_calpain migrates on the gel much more slowly than expected from the predicted 242 kDa molecular weight. We hypothesize that the protein may have intrinsic physical properties that confer anomalous migration, may have an unknown post-translational modification or may be in a stable complex.

### Pf_calpain is detected in the nucleolus

**Fig. 2:**
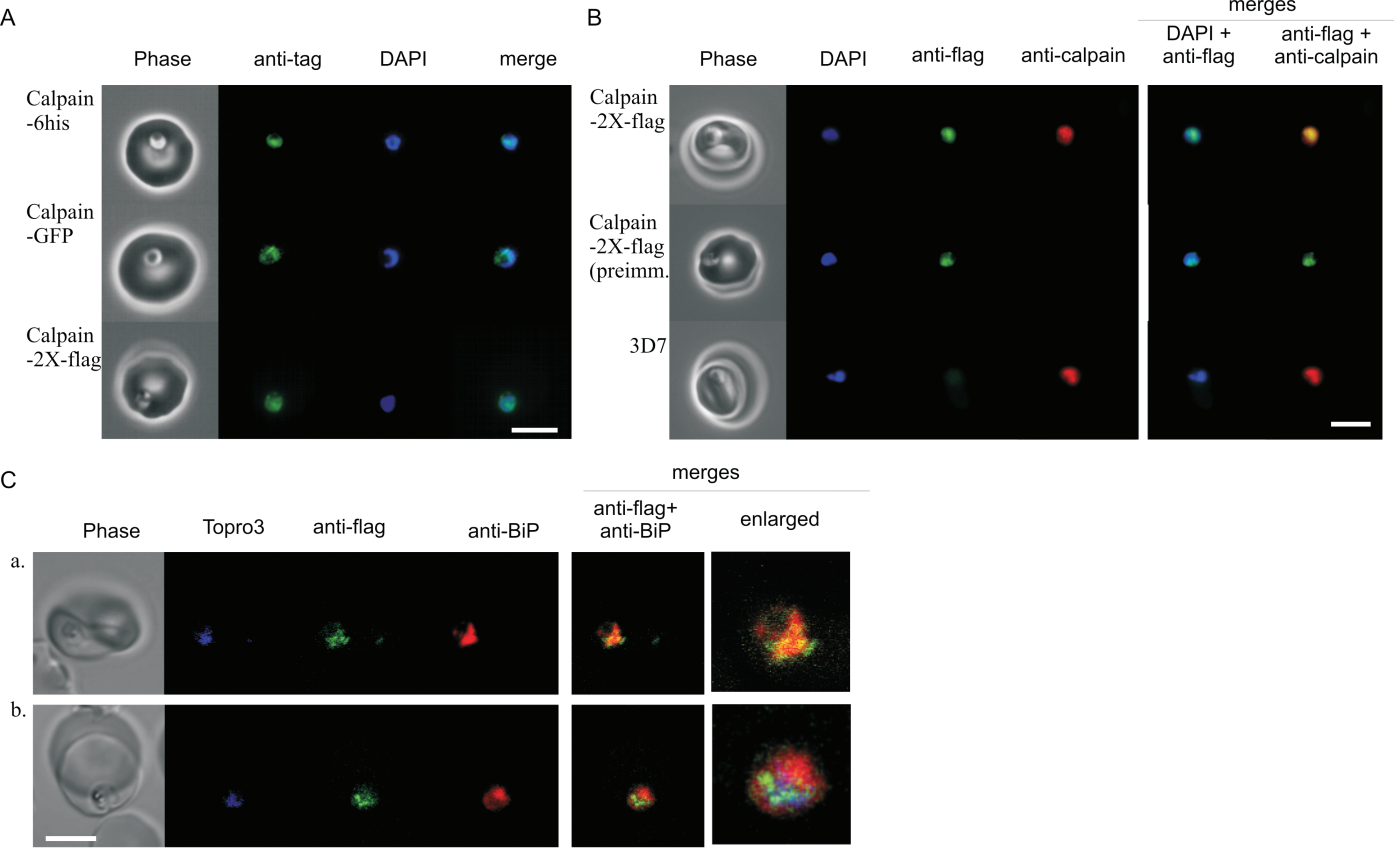
Immunofluorescence localization of calpain. A. Immunofluorescence images of clones expressing calpain-6-his, calpain-GFP and calpain-2×-flag are presented. The images are representative of the overall population and were obtained using tag-specific primary antibodies and a proper secondary antibody conjugated to Alexa-Fluor (AF) 488. The immunofluorescence distribution in all the clones highlights a region closely associated with the nucleus but not completely merging with the DAPI signal. B. Colocalization of fluorescence signal distributions from both anti-flag antibody and anti-calpain antibody with that of a C-terminal peptide of calpain (#35, Fig. S1). Images a and b show the calpain-2×-flag clone stained with monoclonal anti-flag antibody/goat AF488-conjugated anti-mouse antibody and either rabbit anti-calpain #35/goat AF555-conjugated anti-rabbit or pre-immune serum of the same rabbit/goat AF555-conjugated anti-rabbit. The 3D7 parental strain, stained as in a, is positive with anti-calpain #35, but not with the anti-flag antibody. C. The relative distributions of calpain-2×-flag (green channel) and BiP (red channel) signals were analysed by confocal microscopy. Here two representative images show the signal detected in a perinuclear plane (a) and in a nuclear plane (b). The right-most panels present the enlarged merge images. The nucleus in (a) was detected acquiring the Topro3-signal with increased pin-hole that rendered a non-confocal image. Bars, 5 μm.

By immunofluorescence using specific anti-tag antibodies, calpain chimeras (6-his, GFP and 2×-flag) were detected (Fig. 2A). For each we observed a similar fluorescence distribution closely associated with but not completely overlapping that of DAPI-stained nuclei. The fact that all the C-terminal chimeras were detected in similar locations suggests that the localization is independent of the introduced tag and most likely corresponds to the native calpain location. Further, using an anti-calpain peptide antibody, we were able to show that anti-flag and anti-calpain antiserum signals colocalized nicely (Fig. 2B). Colocalization was also observed in the calpain-GFP clone (data not shown). The parental parasite line 3D7 showed a comparable pattern with the anti-calpain antiserum. This confirms that native and tagged calpain have similar locations in the cell. 2×-myc localization was unsuccessful, as tested anti-myc antibodies showed high background above which no significant signal could be detected.

Significant signal with his, GFP and flag tags was obtained only in late ring and early trophozoite-stage parasites, consistent with the peak in concentration of calpain protein (Russo *et al*., 2009). We observed calpain staining that mostly overlapped the DAPI-stained nuclei but in part seemed to be juxta-nuclear. Therefore, we performed confocal analysis of the calpain-2×-flag clone using antibodies for the flag-tag and for BiP, an ER marker (Fig. 2C). Depending on the selected cellular plane, calpain signal either partially colocalized with BiP-stained ER (a) or neatly separated from it (b). Indeed, when the acquired plane cut deep through the nucleus, excluding the BiP signal originating from the top and the bottom of the nuclear body, calpain signal appeared as dispersed spots in the Topro3-stained nuclear mass, far from the surrounding BiP-positive ER. Therefore, we deduced that calpain is found associated with the perinuclear ER, likely the site of its maturation, as well as in a subnuclear location where we detected the most concentrated signal.

**Fig. 3:**
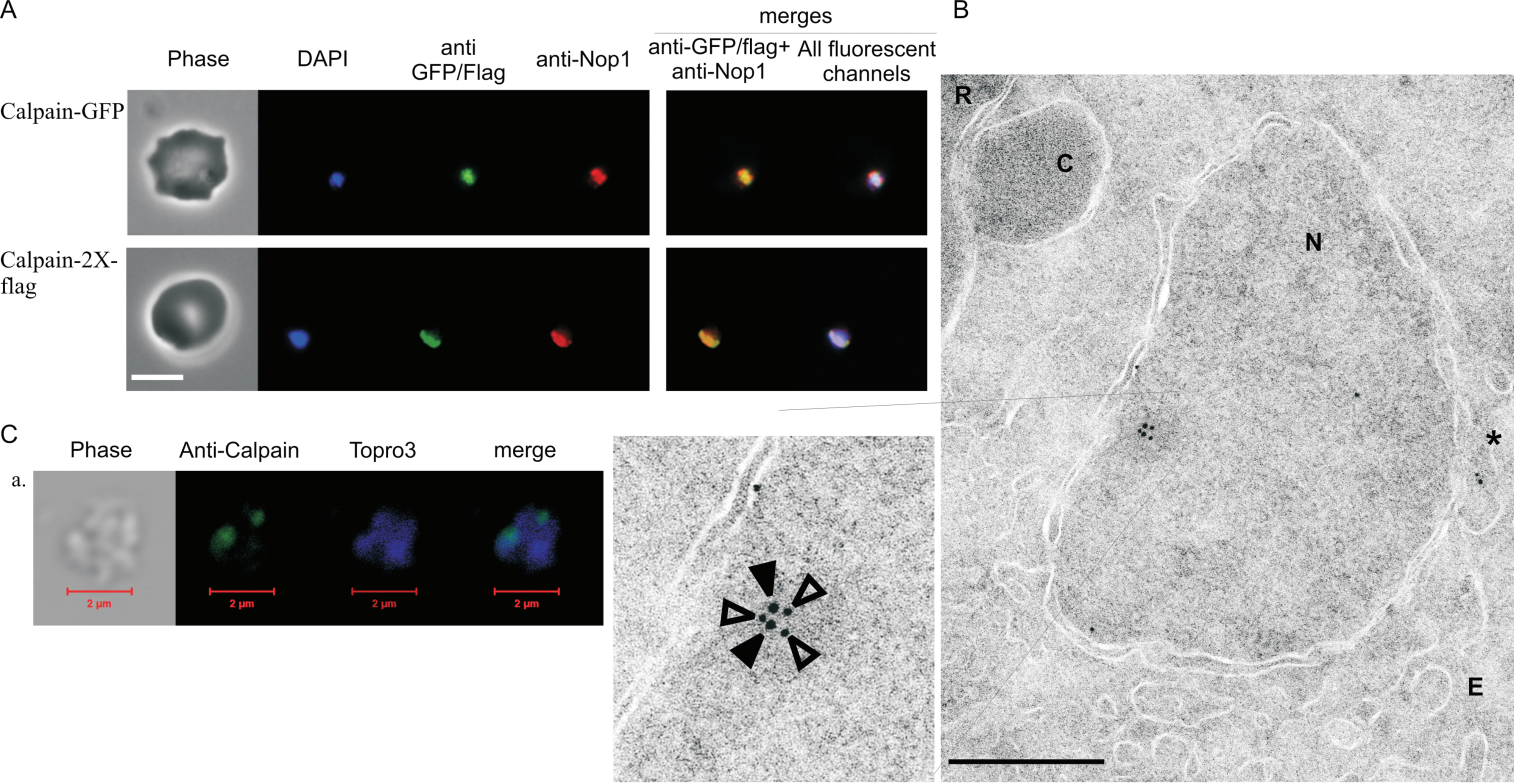
Immunofluorescence and EM analyses reveal that Pf_calpain is nucleolar. A. Colocalization of calpain and Nop1, a nucleolar marker. Using specific antibodies for GFP or for flag epitope in combination with proper AF488-conjugated secondary antibodies, we detected the cellular distribution of calpain in the clones expressing calpain-GFP and calpain-2×-flag in relation to the nucleolar compartment labelling obtained with an anti-Pf_Nop1 serum (red channel). The nuclei are stained with DAPI. Bar, 5 μm. Enlargements of the nuclei are provided in Fig. S3. B. Immuno-EM was done using pre-embedding labelling with anti-flag and anti-Pf_Nop1 after tetanolysin treatment to permeabilize erythrocytes. The image presents the nuclear section of a representative early trophozoite. At the upper left corner are visible the red blood cell (R) and a cytostome (C). The nucleus (N) appears thoroughly circumscribed by its double membrane closely associated with ER (E). Inside the nucleus, labelling due to the immunodetection of calpain-flag (gold particles 12 nm – open arrows in the enlarged panel) and Pf_Nop1 (gold particles 18 nm – solid arrows in the enlarged panel) reveals staining of an electron-dense nuclear subcompartment. Some particles are also found in the perinuclear ER (asterisk). Bars, 0.5 μm. C. Confocal analysis of purified nuclei from 3D7 using anti-calpain antiserum #35. We generally detected one or two spots of approximate diameter of 0.5 μm. Colocalizazion of Nop1 and calpain in an isolated nucleus by confocal analysis is provided in Fig. S3.

The best-characterized subnuclear compartment is the nucleolus, known as the site of rRNA transcription, ribosome and ribonucleoprotein complex assembly and protein sequestration (Birnstiel *et al*., 1963; Maggi and Weber, 2005). We performed dual staining using an antiserum to the well-studied nucleolar marker Pf_Nop1 (Figueiredo *et al*., 2005), in combination with anti-flag or anti-GFP antibodies. The results (Fig. 3A and Fig. S3) show a clear colocalization of Pf_Nop1 with calpain-GFP (a), as well as with calpain-2×-flag (b). Similar colocalization was also obtained using anti-human Nop1 (60% identity to Pf_Nop1, data not shown). Signal with anti-tag antibodies could be detected in late rings and early to mid-trophozoites; a nucleolar focus was seen throughout this developmental period.

In order to further define the calpain-positive subnuclear compartment, our microscopy analysis was completed by two other experiments, electron microscopy (EM) and confocal analysis of purified nuclei. After several inconclusive attempts because of protein scarceness and the small dimensions of the nucleolus, the EM imaging was finally successful when it was performed by using a new method, pre-embedding labelling after tetanolysin permeabilization. Figure 3B shows a representative parasite residing inside a red blood cell (R) from which it is separated by the parasitophorous vacuolar and plasma membranes (PM) that are distinguishable where a cytostome (C) is forming. The parasite nucleus (N) is enclosed in a double membrane that is clearly interrupted in at least three points by nuclear pores and is in continuity with the ER (E). The gold particles indicate the location of the mouse anti-flag (12 nm) and of the rabbit anti-Nop1 (18 nm) antibodies. Both antigens were detected in a subnuclear area that is more electron-dense than the rest of the nucleus. We believe this to be the nucleolus. There were also some particles outside the nucleus (asterisk) in the juxta-nuclear ER, as expected.

Confocal analysis using anti-calpain antiserum was performed on isolated parasite nuclei (Fig. 3C). Using this technique we were able to significantly enhance calpain signal through improved antibody accessibility and decreased light-interacting barriers. In all the pictures collected, the calpain-positive subnuclear compartments were one or two discrete areas inside the DAPI-stained nuclear mass, resembling kidney-shaped or circular spots, whose diameter averaged about 0.5 μm. We detected no difference between the two analysed strains, 3D7 and HB3 (data not shown). Calpain signal colocalized with that of Nop1 (Fig. S3). Pf_calpain is the first calpain to be detected inside the nucleolus.

### Pf_calpain domain composition

By phylogenetic analysis, we showed that Pf_calpain is a distinct type of calpain (Russo *et al*., 2009). The most dramatic difference from the rest of the superfamily resides in the N-terminal domain, which spans half of the protein and has no significant homology to any other known protein or recognizable function (Russo *et al*., 2009). Calpains from all *Plasmodium* species have a similar large N-terminal domain. Orthologues to Pf_calpain from other *Plasmodium* species (Table S1) were aligned using ClustalX (Chenna *et al*., 2003) and Jalview (Clamp *et al*., 2004) (Fig. S2). We finely analysed the alignment to detect conserved motifs and subdomains that could be responsible for the unusual nucleolar localization.

**Fig. 4:**
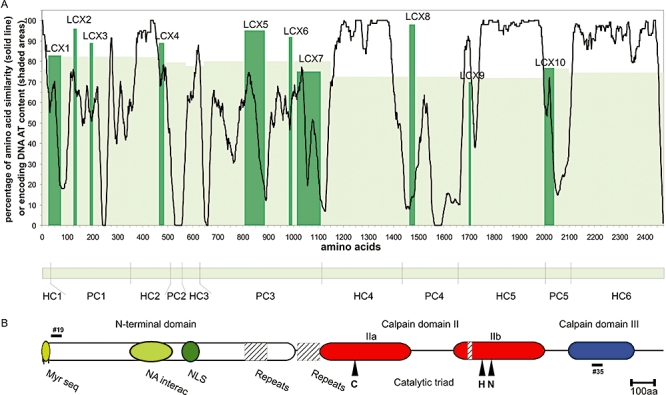
Features and conservation of *Plasmodium* calpains. A. Calpain conservation in *Plasmodium* species and Pf_calpain AT content. In the graph, the *x*-axis shows amino acid position along the alignment of Fig. S2. The black line is the percentage of similarity among calpains encoded by *P. falciparum*, *reichenowi*, *gallinaceum*, *yoelii yoelii*, *berghei*, *chabaudi*, *knowlesi* and *vivax* (Table S1). The degree of conservation was calculated in a 20-amino-acid sliding window throughout the aligned calpain sequences. AT percentage of each *P. falciparum* subdomain is graphically represented by the green shading. The relative positions of nine low-complexity regions (LCX 1–9) detected in 3D7, are indicated as dark green bars, the height of which indicates AT% content of the corresponding DNA fragment. LCXs 5, 7 and 9 correspond to DNA repeats. Six highly conserved (HC 1–6) and five poorly conserved (PC 1–5) regions are depicted in the lower green bar, in scale to the graph. B. Diagram of Pf_calpain domains and subdomains. Regions are in scale to the graph in (A). The highly conserved regions in the N-terminal domain are, in order, a conserved myristoylation consensus sequence (Myr seq), a subdomain common to some nucleic acid-interacting proteins (NA interac) and a putative nuclear localization signal (NLS). The central domain has homology (*E*-value 9.59 e^−34^) to calpain catalytic domains (domain II) containing the catalytic triad C, H and N (black arrows indicate their relative positions). The C-terminus shows homology (*E*-value 1.84 e^−3^) to calpain domain III. Positions of repeats (shaded boxes) and peptides used to generate rabbit antisera #19 and #35 (bars) are shown.

The averaged overall homology among *Plasmodium* calpain amino acid sequences is 67% and the identity 55%. However, the homology is not evenly distributed along the protein. Therefore, in order to analyse the conservation distribution, we designed a PERL program that calculates homology in a sliding 20-amino-acid window. The result shows that Pf_calpain has six highly conserved regions (HC) interspersed with five poorly conserved ones (PC) (Fig. 4A).

The PC areas are not explained simply by higher AT content, or by the presence of low complexity regions (LCX) (Wootton and Federhen, 1996), and a deeper analysis revealed that they are conserved in closely related species (Fig. S4). In *P. falciparum* isolates there are very few non-synonymous single nucleotide polymorphisms and these are excluded from HC regions. Our analysis indicates a high overall conservation of the *Plasmodium* calpains and particularly of their subdomains.

All the HC regions of Pf_calpain seem to correspond to functional subdomains (Fig. 4B). There is a myristoylation consensus sequence (HC1), a motif not previously reported but conserved in proteins that interact with nucleic acids (HC2), a putative nuclear localization signal (HC3), calpain catalytic subdomains IIa and IIb (HC4 and HC5) and calpain domain III (HC6). HC2 is a particularly intriguing subdomain, highly conserved in alveolates calpains but not annotated in SMART, Pfam, ProDom, InterPro and Prosite databases. By using the NCBI pBlast for protein and peptides (Altschul *et al*., 1990), we found similarity to a number of hypothetical *Plasmodium* proteins and to a group of proteins predicted to interact with nucleic acids in various organisms (Table S2). Although we can speculate that this region may be involved in nucleic acid interactions, further analysis is needed to define its actual functional role.

HC4 has homology to calpain domain IIa that includes the catalytic Cys (1053) (*E*-value 4 e^−28^) and HC5 matches calpain domain IIb that contains the catalytic His (1389) and Asn (1409) (*E*-value 1 e^−20^). The loop that divides the two subdomains is predicted not to disrupt the proteolytic activity, as separating loops have been observed in other calpains, like calpain 3 (Sorimachi, 2004). Indeed, the available calpain three-dimensional structures reveal that domains IIa and IIb, folding as independent units, are physically separated (e.g. 1KFU, PDBbank). Associating together, domains IIa and IIb generate a catalytic pocket where the cysteine faces the other two catalytic triad residues (Strobl *et al*., 2000). Lastly, HC6 has homology, although more distant (*E*-value 1.84 e^−3^), to calpain III domains.

### A short sequence from Pf_Calpain is sufficient to confer nucleolar localization

Pf_calpain subdomain HC3 contains a predicted bipartite nuclear localization signal of the kind first found in *Xenopus* nucleoplasmin (Robbins *et al*., 1991). This was recognized by PSORT II (Nakai and Horton, 1999), which gave a nuclear prediction with a reliability of 94.1% and a k-nearest neighbour score of 73.9%. We also analysed the sequence using ‘PredictNLS’ program (Nair *et al*., 2003), which bases its prediction on database annotations, functional information and a system of neural networks. PredictNLS identified two potential NLS motifs, one right downstream of the myristoylation motif in HC1 and the other one in HC3. The first one could be summarized as either [KR]KRKK or KxKxKxxxxxRKK consensus sequences and it is similar to the classical SV40 large T antigen NLS (Kalderon *et al*., 1984). These consensus sequences are found in 50 and 11 proteins respectively, and all of them are nuclear. The other motif in subdomain HC3 can be annotated as [RK]{3,}?x{8,16}[RK]{4,}?, [KR]{4}x{20,24}K{1,4}xK or R[RK]x[KR]x[RK]{2,}?[DE]. Such motifs are found in 193 (97.9% nuclear), 161 (97.5% nuclear) and 132 (all nuclear and 90.15% of them bind the DNA) proteins, respectively. As the classical myristoylation sequence is often associated with a polybasic stretch that stabilizes phospholipids/protein interaction (Resh, 1999), we discounted the recognition of this region as an NLS and concentrated our investigation on the putative NLS in HC3.

**Fig. 5:**
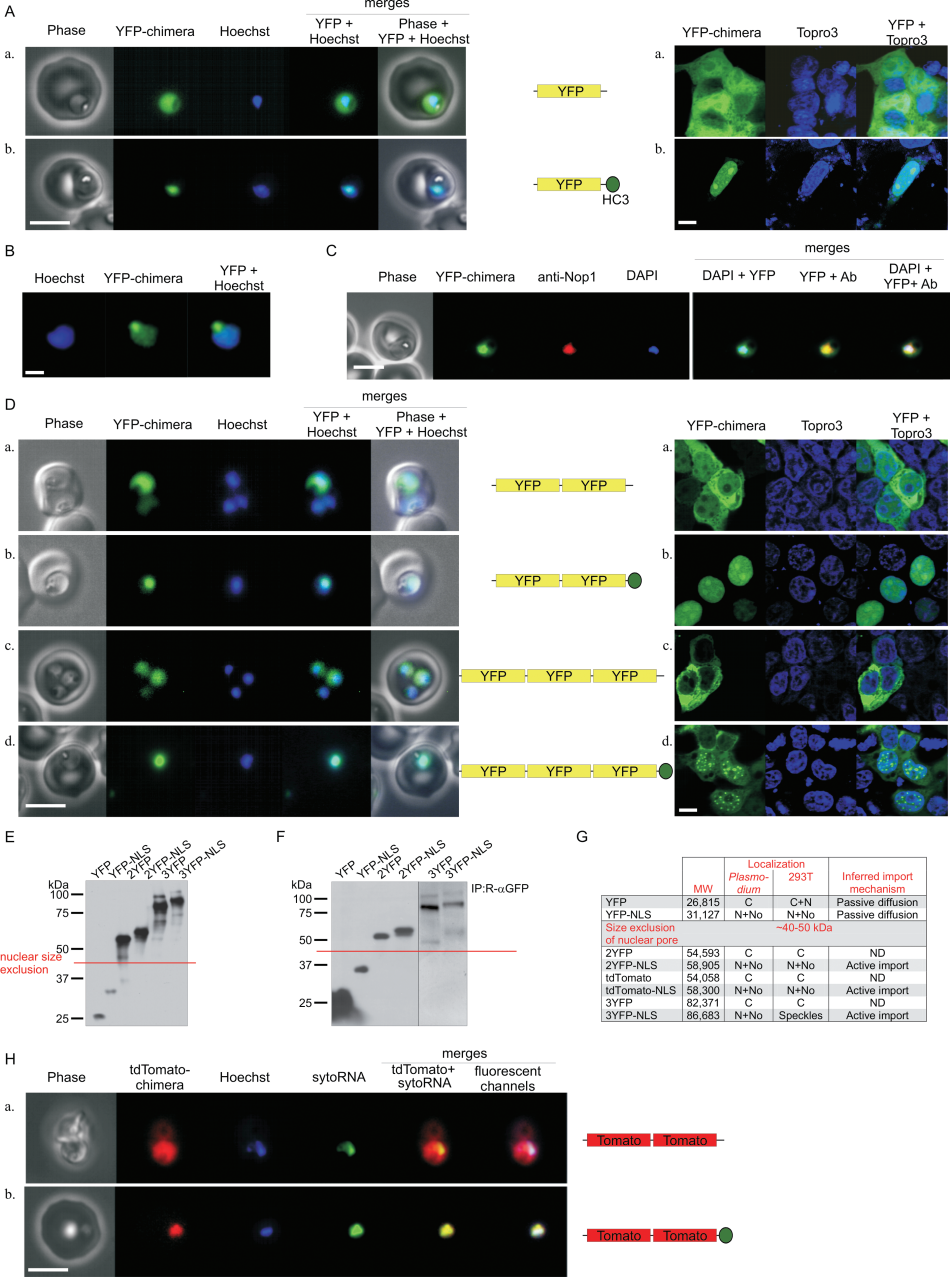
Localization of YFP-HC3 in transfected parasites and mammalian cells. A. Single YFP chimera localization. Left, *P. falciparum* (bars, 5 μm); right, human 293T cells (bars, 1 μm). Between the panels are schematics of the constructs expressed. Green circle, HC3 subdomain. B. Enlargement of nuclei of fixed *P. falciparum* cells expressing YFP-NLS, showing discrete subnuclear spots. Bar, 1 μm. C. Colocalization of *P. falciparum* YFP-HC3 fusion (YFP) with anti-Pf_Nop1. DAPI staining is also shown. Merges are to the right. D. Multi-YFP chimera localization. Cells and schematics as in (A). Enlargements of the nuclei are provided in Fig. S3. E and F. Expression pattern of the YFP chimeras used in mammalian cells (E) and *P. falciparum* (F) by Western blot using anti-GFP antibody. G. Tabulation of construct sizes, putative nuclear entry mode and significant localization. C, cytoplasmic; N, nuclear; No, nucleolar. H. Live *P. falciparum* colocalization of Td-Tomato (a), Td-Tomato-HC3 chimera (b) with sytoRNA green, a dye to detect the nucleolus in live cells. Bar, 5 μm. Enlargements of the nuclei are provided in Fig. S3.

We generated an YFP chimera carrying 105 bp of HC3 sequence. This chimera was cloned into two different vectors, one suitable for *Plasmodium* transfection and the other for mammalian expression. Live epifluorescence images of transfected *Plasmodium* and confocal pictures of fixed transfected mammalian cells are shown in Fig. 5A. The HC3 sequence drove the chimera into the nucleus and concentrated it in nuclear subregions of *Plasmodium* and mammalian cells (Fig. 5A and B). The subnuclear spots colocalized with the nucleolar marker Nop1 (Fig. 5C).

Molecules with mass less than the nuclear pore exclusion limit of ∼40–50 kDa may enter the nucleus by passive diffusion and could accumulate in this location by trapping once there (Terry *et al*., 2007). Therefore, in order to assess whether the HC3 region is an actual NLS promoting active import into the nucleus, we increased the size of our chimeras by using multiple YFP domains. For all chimeras and in both cell types we detected nuclear fluorescence (Fig. 5D and Fig. S3) and full-length proteins (Fig 5E and F). In *Plasmodium* there was nuclear and nucleolar localization for the double and triple YFP chimeras, while for mammalian cells even though the triple YFP-NLS reached the nucleus it did not seem to enter the nucleoli but gave a speckle-like distribution. Perhaps there is nucleolar intolerance to or exclusion of such a chimera. Our results, summarized in Fig. 5G, showed that Pf-calpain HC3 has a functional NLS that contains an embedded nucleolar localization signal (NoLS), able to promote nuclear import and nucleolar concentration.

Similarly, when calpain NLS was fused to td-Tomato protein (Shaner *et al*., 2004), which is similar in size to 2YFP, the chimera was also driven to the nucleus and concentrated in the nucleolus (Fig. 5H and Fig. S3). This last chimera allowed visualization of the nucleolus in live cells, using SytoRNA green and the calpain NLS chimera distribution. The reporter and the RNA dye signals colocalize when td-Tomato is fused to the calpain NLS and both of them partially colocalize with Hoechst-stained DNA, defining a clear round peripheral district that corresponds to the nucleolus.

### HC1 has an acylation motif that confers membrane localization

**Fig. 6:**
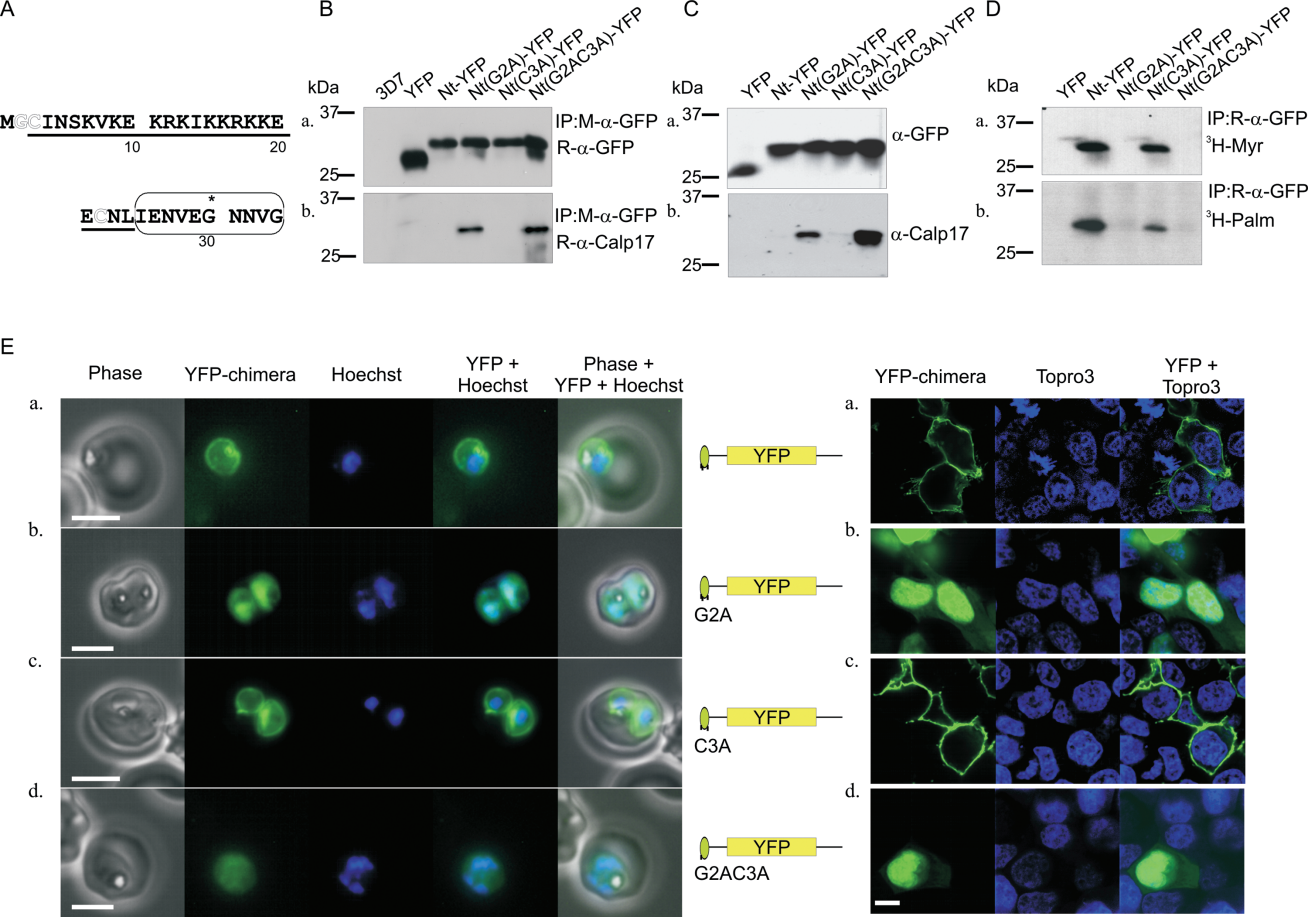
HC1 is membrane anchored as a consequence of acylation. A. The sequence of HC1. The amino acids predicted to be modified by myristate (Myr) and palmitate (Pal) are indicated by outlined letters. The bar indicates the peptide used to generate anti-calpain antiserum #17. Amino acids included in the longer version of the calpain N-terminal HC1 chimeras (NtL) and excluded from the shorter version (Nt) are encircled. Asterisk, potential cleavage site. B. Analysis of constructs in *P. falciparum*. Stable lines of transfected parasites were generated, using plasmids that drive the episomal expression of chimeras carrying wild-type HC1 or mutants in amino acids 2 and 3. Immunoprecipitated chimeras were analysed by Western blot using rabbit anti-GFP antibody (a) and, after stripping of the membrane, rabbit-anti-calpain antiserum #17, which recognizes only the unmyristoylated constructs (b). C and D. Analysis of constructs in mammalian cells. 293T cells were transfected with plasmids encoding each of the chimeras as in (B). The cells were grown in normal (C), or labelling media (D). Cell lysates were analysed by Western blotting (C) using anti-GFP (a) to monitor expression levels or anti-calpain antiserum #17 (b). Alternatively, cells were labelled with ^3^H-myristate (a) or ^3^H-palmitate (b), lysates were immunoprecipitated with anti-GFP antibody and fluorography was performed (D). ^3^H-palmitate but not ^3^H-myristate label was sensitive to hydroxylamine (not shown). E. Fluorescence of live transfected *P. falciparum* (left; bars, 5 μm) and fixed human 293T cells (right; bar, 1 μm). Schematics of the constructs are shown. Light green oval, HC1 subdomain. Below each schematic is the mutation constructed. Images are labelled as in Fig. 5. NtL chimeras are shown in Fig. S5.

While Pf_calpain HC3 is sufficient to drive the protein to the nucleus/nucleolus, HC1 potentially confers membrane localization to the protein, as it is predicted to be lipid-modified. HC1 corresponds to the N-terminal consensus sequence, MGxxxS, which is cotranslationally recognized, freed of the first methionine by a methionine aminopeptidase, and irreversibly myristoylated on the amino group of the glycine in other systems, as well as in *P. falciparum* (Moskes *et al*., 2004). In addition, if cysteines are present in the vicinity of Gly2, upon membrane association the protein is likely to be post-translationally palmitoylated on the thiol group. The Pf_calpain N-terminal sequence contains a cysteine in position 3 (Fig. 6A), one of the most common palmitoylated sites that defines the type IV dually acylated protein group (Resh, 1999).

We generated YFP-chimeras carrying HC1 and made mutants, in which Gly2 and Cys3 were replaced by alanines (Fig. 6). Both *in vitro* (data not shown) and *in vivo* expression of the chimeras showed that the consensus sequence is myristoylated as long as the Gly is unperturbed, while Cys3 is dispensable. The acyl modifications were detected not only by radioactive labelling (Fig. 6D panel a), but also using a specific rabbit antiserum (Fig. 6B and C), raised against a calpain N-terminal peptide (bar in Fig. 6A). This reagent showed specificity for the un-acylated N-terminus, rendering a positive signal in all G2A mutants in both cell types.

The wild-type HC1 chimera was also palmitoylated (Fig. 6D panel b) and localized neatly along the PM, in *Plasmodium* as well as in mammalian cells (Fig. 6E panel a, EM in Fig. S5). The PM localization was independent of the presence of Cys3, whereas cytoplasmic localization was observed with G2A mutants. These last mutants, especially in the mammalian cells, also gave nuclear signal. This is probably due to the polybasic stretch of amino acids present in HC1, which may function as an artefactual nuclear signal, not being involved any more in the stabilization of membrane/myristoyl-protein binding.

**Fig. 7:**
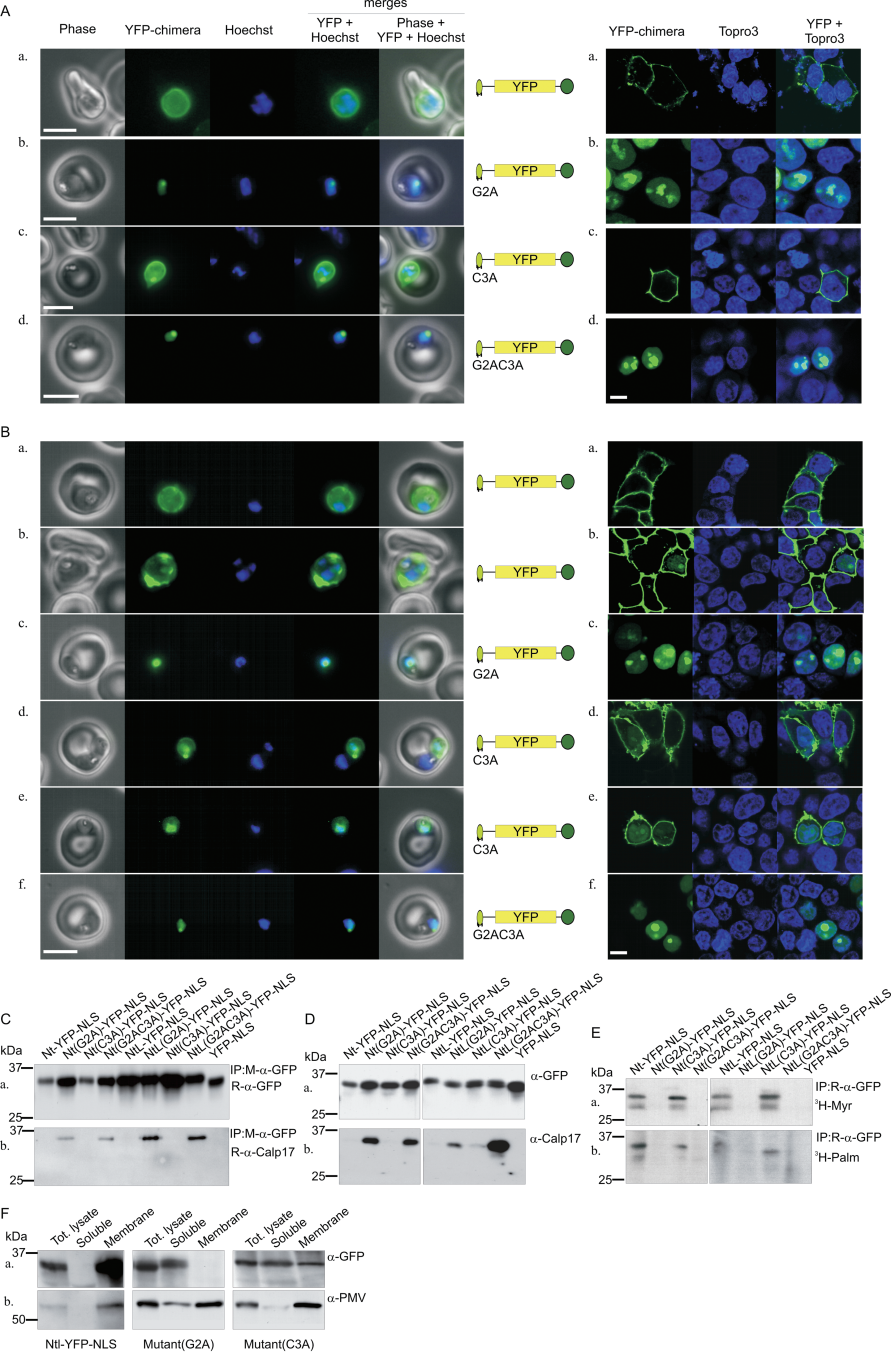
Interaction of HC1 and HC3 regions in reporter targeting. A and B. Fluorescence of live *P. falciparum* (left; bars, 5 μm) and fixed human 293T cells (right; bar, 1 μm). As in Figs 5 and 6, schematics of the constructs and mutations are shown and images are labelled at the top. Chimeras shown have the short HC1 version (Nt) (A) or long one (NtL) (B). C. Analysis of expressed constructs in *P. falciparum*. As in Fig. 6, chimeras, immunoprecipitated from stably transfected parasites, were analysed by Western blot using rabbit anti-GFP antibody (top) and, after stripping of the membrane, rabbit-anti-calpain antiserum #17 (bottom). D and E. Analysis of constructs in mammalian cells. 293T cells were transfected with plasmids encoding each of the chimeras as in (C). The cells were grown in normal (D), or labelling media (E). In (D), cell lysates were analysed by Western blotting using anti-GFP (top) to monitor expression levels or anti-calpain antiserum #17 (bottom). E. Alternatively, lysates were immunoprecipitated with anti-GFP antibody and fluorography was performed. F. Cellular fractionation analysis of Ntl-YFP-NLS and its two HC1 mutants, G2A and C3A. The distribution of the small chimeras (panel a) and Plasmepsin V (PMV), a membrane marker (panel b) in the total lysate (1/2 vol.), soluble (1 vol.) and membrane fractions (1 vol.) is shown.

No palmitate labelling was detected in G2A mutants (Fig. 6D panel b), as in this type of motif palmitoylation depends on prior myristoylation (Resh, 1999). However, when Cys3 was mutated to Ala, palmitate labelling of the chimeras was not abolished but only reduced, indicating the presence of a second palmitoylation site (see below). These results suggested that HC1 contains a functional acylation motif that anchors the protein to membranes. Further evidence is provided below (Figs 7 and 9).

#### 

##### 

###### Interaction of HC1 and HC3 targeting signals

As Pf_calpain, which contains both HC1 and HC3, is detected in the nucleolus, to study the interaction of these two signals we sandwiched YFP between them. These chimeras showed a PM distribution (Fig. 7A panel a) and were both myristoylated and palmitoylated (Fig. 7C–E). Whenever the G2A mutation was introduced, clear nucleolar localization was seen (Fig. 7A). When Cys3 was mutated, the chimeras remained predominantly PM-localized.

In these chimeras HC1 (Nt) has a dominant effect in the targeting with respect to the HC3 NLS. However, the fact that full-length Pf_calpain reaches the nucleolus implies that some regulatory mechanism exists to reconcile the two contrasting targeting signals and overcome the dominant effect of the membrane anchor. We submitted Pf_calpain sequence to SignalP 3.0 (Emanuelsson *et al*., 2007) and found a potential proteolytic site in the calpain N-terminus at position 29 (asterisk in Fig. 6A). Therefore, we fused a longer version of HC1, NtL, to YFP (Fig. 6A). No processing was detected and, localization of the chimeras carrying only the HC1 was identical for both versions (Nt chimeras in Fig. 6 and NtL chimeras in Fig. S5). We also expressed the Pf_calpain N-terminus up to the start of HC3 (∼420 aa) fused to GFP. This chimera, still unprocessed, remained PM-localized (data not shown).

Surprisingly, when the NtL-YFP chimera was fused to HC3, although no significant N-terminal cleavage was detected (Fig. 7C–E panels a), substantial differences in the localization were seen compared with the Nt chimeras. We observed that the wild-type NtL chimera primarily has a PM distribution (Fig. 7B panel a) and in occasional cells (less than 1%) a dual localization (PM and nucleus) (panel b). Interestingly, while G2A mutants showed nucleolar signal as expected (c and f), the C3A mutant appeared able to enter the nucleus (d and e), even though still myristoylated (Fig. 7C–E). About 5% of cells still showed PM signal. The fact that the longer version of the N-terminus, but not the shorter, can be released from the membranes and move into the nucleus suggests that the extra ten amino acids confer the sequence information or the physical properties needed.

### Role of palmitoylation status in calpain localization

To confirm that the PM-localized chimeras were truly in the membrane and the nuclear ones soluble, we performed cellular fractionation (Fig. 7H). The wild-type NtL chimera fractionated with the membranes and the G2A mutant was soluble. The C3A mutant was partially membrane-bound and partially soluble, consistent with the microscopy.

**Fig. 8:**
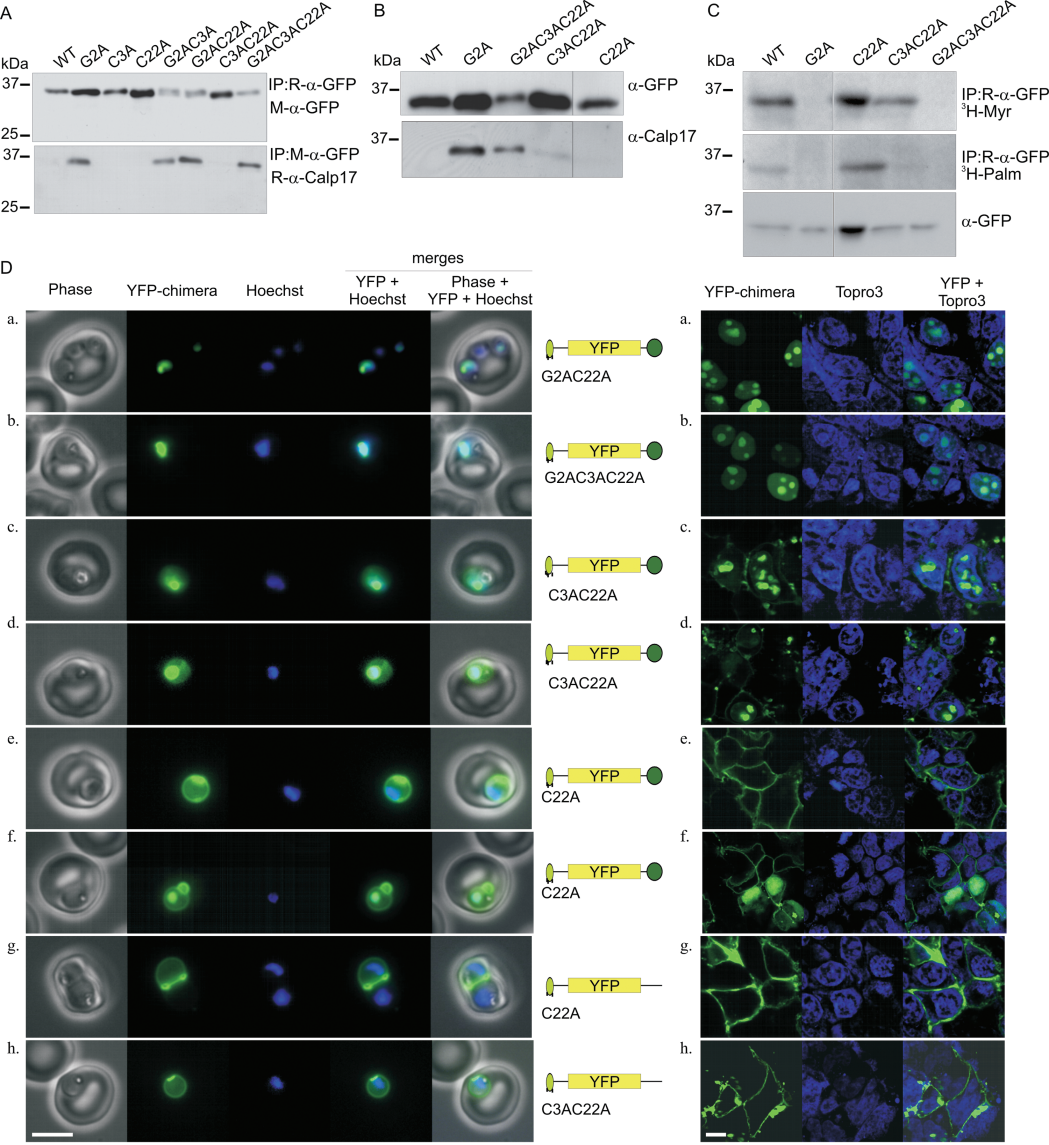
Palmitoylation occurs at cysteines 3 and 22. Analysis of constructs expressed in *P. falciparum* (A) and mammalian cells (B and C). Cell lysates were processed as in Fig. 6. (D) Fluorescence of live parasites (left; bar, 5 μm) and fixed 293T cells (right; bar, 1 μm). As in Fig. 7, schematics of the constructs and mutations are shown and images are labelled at the top. Chimeras shown have the long HC1 version (NtL).

Because of the dual cellular distribution and the fact that some palmitate labelling was still detected in the C3A mutant, we examined a second cysteine downstream at position 22 (Fig. 6A). A new set of mutations was analysed (Fig. 8). While the C22A mutant was still labelled with palmitate, the double mutant C3A/C22A was not (Fig. 8A–C), suggesting that Pf_calpain gets palmitoylated at two different sites, Cys3 and Cys22. The chimeras concentrated in the nucleus and nucleolus whenever myristoylation was prevented (Fig. 8D panels a and b), as before, and when the palmitoylation was completely abolished (panels c and d). Mutation of C22 alone resulted mostly in PM association (panel e) although in a significant portion of cells (∼40%) some nuclear concentration was seen (panel f). In contrast to the C3A mutant (Fig. 7B), no C22A cells expressing only nuclear fluorescence were observed. This suggests that the palmitoylation at position 3 has a principal role in trapping the protein in the membrane. When we removed HC3 from the chimeras, both C22A and C3A/C22A mutants remained PM-associated (Fig. 8D panels g and h), indicating that the myristoyl moiety is sufficient for PM association of HC1. We believe that HC3 is able to target myristoylated protein to the nucleus because binding of myristoylated peptides to the membrane is weak and reversible in the absence of palmitate anchors (Peitzsch and McLaughlin, 1993).

**Fig. 9:**
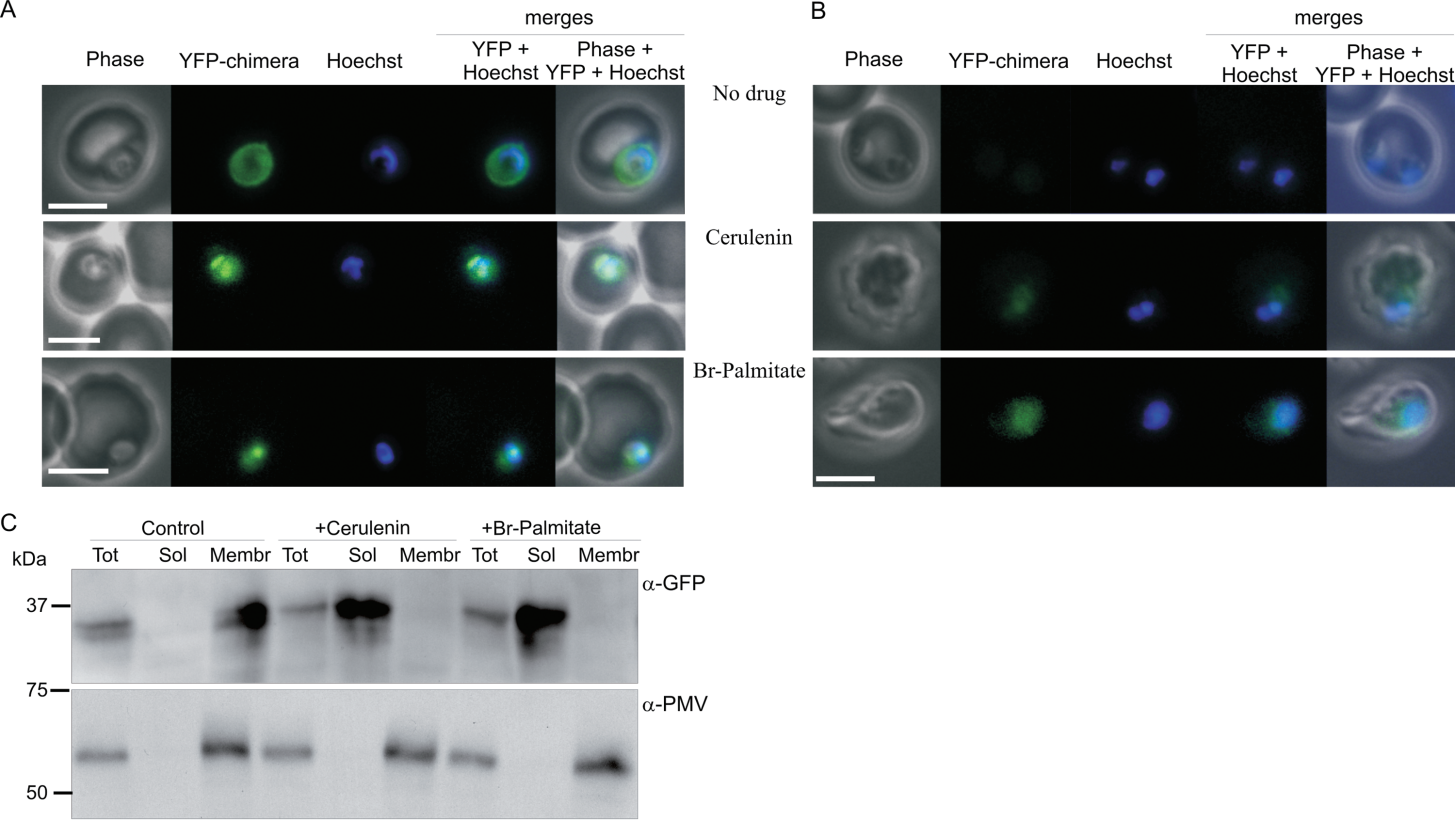
Localization of wild-type HC1-YFP-HC3 and full-length calpain-GFP in parasites treated with palmitoylation inhibitors. Live fluorescence of stably transfected *P. falciparum* episomally expressing NtL-YFP-NLS (A) and integration clone C3 expressing calpain-GFP (B). Images are labelled at the top for phase contrast, YFP, nuclear stain and merged channels. No drug controls; 2.5 μg ml^−1^ cerulenin; 10 μg ml^−1^ 2-Bromo-palmitate are shown. Incubations were carried out for 20 h. Bars, 5 μm. (C) Cellular fractionation analysis of palmitoylation inhibitor-treated NtL-YFP-NLS. The distribution of the small chimera (panel a) and Plasmepsin V (panel b) in the total lysates (Tot, 1/2 vol.), soluble (Sol, 1 vol.) and membrane fractions (Membr, 1 vol.) is shown.

To further explore the role of palmitoylation in calpain localization, we treated parasites expressing the HC1-YFP-HC3 chimera with palmitoylation inhibitors. After ∼27 h incubation, parasites were assessed by live fluorescence microscopy (Fig. 9). An untreated control showed a PM distribution, as expected, while in cells treated with either cerulenin or 2-Bromo-palmitate the signal concentrated in the nucleus/nucleolus. Cellular fractionation confirmed that the palmitoylation inhibition caused the chimera to shift from membrane-bound to soluble form. The full-length calpain-GFP integrant clone C3 was treated similarly. The control culture displayed no detectable green fluorescence because the endogenous expression level is too low to be visualized in live cells. In contrast, inhibitor-treated cells displayed appreciable fluorescence with a nuclear/nucleolar pattern. We suggest that impairment of palmitoylation leads to accumulation of calpain in the nucleus to a level at which it can now be detected.

## Discussion

We have found that Pf_calpain is a nucleolar protein and, using an informatics scan, have identified motifs that determine its localization in *P. falciparum*. Members of the calpain superfamily are found from bacteria to protozoa, from plants to mammals (Croall and Ersfeld, 2007). They are extremely divergent and have in common the catalytic core that is found in combination with different ‘modules’ or domains. Specific cellular functions depend on these domains, which modulate enzymatic activity, substrate specificity and cellular location (Sorimachi *et al*., 1997; Goll *et al*., 2003). Pf_calpain has a large N-terminal domain. *Plasmodium* proteins are often larger than their orthologues, mostly due to insertion of low-complexity sequences. In Pf_calpain, although low-complexity regions do exist, they are a minor component of the unusual extension that spans half of the entire protein. This N-terminus contains three HC regions. These correspond to a putative myristoylation motif, a previously unidentified but conserved domain common to some nucleic acid-interacting proteins and a putative NLS. *Drosophila melanogaster* Sol is the only other calpain where we recognize a putative myristoylation signal, but its functionality has yet to be established. The finding of an NLS is also new, although evidence for nuclear translocation of some other calpains was recently reported (Gil-Parrado *et al*., 2003; Tremper-Wells and Vallano, 2005).

To study Pf_calpain *in vivo* we generated 3D7 clones expressing different C-terminal tagged versions by endogenous locus modification. This enzyme is expressed at such low levels that its detection was challenging at each step. However, optimizing classical techniques, we were able to visualize Pf_calpain on WBs, as well as through fluorescence and EM. Our tagged and native calpain versions had a similar dual distribution: perinuclear ER and nucleolus. Pf_calpain is the first reported nucleolar calpain.

We identified a 35-amino-acid sequence that drove an YFP reporter not only inside the nucleus but also into the nucleolus. Interestingly, this targeting signal worked efficiently in mammalian cells, suggesting a conservation of targeting information between host and parasite. Proteins are targeted to the nucleolus by multiple mechanisms and no unique features defining a NoLS have been recognized (Meng *et al*., 2007). However, frequently the NoLS appears to overlap the NLS, as is the case for Pf_calpain. We noticed a striking similarity of the Pf_calpain HC3 to the NLS of human nucleolar RNA helicase (RH II/Gu) (data not shown), but the critical residues that determine nucleolar concentration remain to be identified. Other proteins have unexpectedly been localized to the *Plasmodium* nucleolus (Figueiredo *et al*., 2005), although no common motif is apparent.

HC1 has a myristoylation consensus sequence, but this is not an indication of membrane localization *per se* because myristate has an apparent Kd of ∼0.1 mM for the binding to a phospholipid bilayer (Peitzsch and McLaughlin, 1993), insufficient to stably anchor the protein to membranes (Resh, 2004). However, Pf_calpain has two of the five characterized ‘co-occurring factors’ that stabilize membrane attachment of myristoylated proteins (Maurer-Stroh *et al*., 2004): a patch of positive charges that interact with the negatively charged phospholipids downstream of the consensus sequence and a palmitoylation site in close proximity to the myristate. Therefore, it was not unexpected that a chimera carrying the Pf_calpain N-terminus would bind to membranes, as we observed in *Plasmodium* and in mammalian cells. We demonstrated that HC1 is myristoylated and dually palmitoylated, and even when palmitoylation is abolished by double cysteine mutation, the HC1-YFP chimera remains membrane-associated.

Having found two potentially conflicting targeting signals, HC1 and HC3, we assessed location of chimeras containing both subdomains. We found that such constructs localized to the PM unless palmitoylation was blocked by mutation of the cysteines or by treatment with palmitoylation inhibitors. In these cases nuclear/nucleolar localization was seen. The size of the HC1 domain made a difference. In versions where an extra ten amino acids were added to the end (NtL), occasional cells showed nuclear signal and when one or both cysteines were mutated, a substantial amount of reporter trafficked to the nucleus/nucleolus. We suggest that HC1 extension stabilizes the membrane-free form of the myristoylated protein; subsequently, HC3 further shifts the equilibrium of weak membrane association by removing membrane-released protein to the nucleus.

Strong stabilization of membrane binding through dual palmitoylation prevented nucleolar concentration of the HC1-HC3 chimeras. The membrane-nuclear shuttling of some proteins, for example, R7BP (Drenan *et al*., 2005), phospholipid scramblase 1 (Wiedmer *et al*., 2003), or kinetic bilayer trapping of others, for example, fyn (Shahinian and Silvius, 1995), can be regulated by palmitoylation. This is a reversible acylation and could be important for the regulation of Pf_calpain localization as well, as suggested by the palmitoylation inhibitor experiment.

Our data do not discriminate whether the nucleolus is used for Pf_calpain sequestration or as the final destination where it exerts its activity. Protein precedents exist for either model: sequestered proteins, like Mdm2 (Maggi and Weber, 2005), and nucleolar proteases, like Smt3-specific isopeptidase (Nishida *et al*., 2000) and SUMO-specific proteases SENP3 and 5 (Di Bacco *et al*., 2006; Gong and Yeh, 2006). Calpains have been shown to accumulate away from their site of action (m-calpain, after cytosolic activation moves to the PM) (Saido *et al*., 1994). The ER pool of calpain could represent a biosynthetic site, a degradative site or the site of action. We favour the nucleolus as the active destination for Pf_calpain. The nucleolus, known as the site of rRNA transcription, ribosome and ribonucleoprotein complex assembly and protein sequestration, has also been shown to be involved in cell cycle regulation (Birnstiel *et al*., 1963; Maggi and Weber, 2005). It is therefore of interest that knockdown of Pf_calpain affects asexual cell cycle progression (Russo *et al*., 2009). Interactome data may also point to a functional role in the nucleolus. By two-hybrid screening, the 40S ribosomal protein S27 appears to reproducibly interact with Pf_calpain (LaCount *et al*., 2005).

We have identified Pf_calpain as a nucleolar protein that is targeted by a phylogenetically conserved mechanism and is regulated by myristoylation-dependent palmitoylation. Whether the protein is released from the membrane by depalmitoylation, allowing nucleolar trafficking, or is initially trafficked to the nucleolus in a non-palmitoylated state and later removed from that site by palmitoylation trapping remains to be determined.

## Experimental procedures

### Cell culture

*Plasmodium falciparum* 3D7 was cultured as previously described (Drew *et al*., 2008). Human kidney cell line 293T (CRL-11268) was maintained as recommended by the ATCC. Cultures to be treated with palmitoylation inhibitors were grown with 0.05% AlbumaxII instead of 5%.

### Transfections and selection

Eighty microlitres of packed RBC was transfected by electroporation with ∼100 μg of purified vector (Fidock and Wellems, 1997), and then infected at 0.5% parasitemia. Positive selection was initiated after 48–72 h using 10 nM WR99210. Transfectants with episomally maintained plasmid were cultured under drug pressure. Integration required two to three rounds of drug cycling (Wu *et al*., 1996).

Mammalian cells were transfected with Lipofectamine 2000 (Invitrogen) by the manufacturer's instructions. After overnight incubation and an additional 8 h in fresh medium, cells were harvested.

### Phylogenetic analysis

The sources of calpain sequences are in Table S1. Alignments were generated with the ClustalX program (Chenna *et al*., 2003) (penalties: pair gap, 50; gap extension, 2.5; multialignment gap opening, 25) and revised using Jalview (Clamp *et al*., 2004).

Database searches were performed using blast (Altschul *et al*., 1997). Pf_calpain was analysed with PsortII (Nakai and Horton, 1999), Myristoylator (Bologna *et al*., 2004) and PredictNLS (Nair and Rost, 2004). To analyse the conservation distribution we used a PERL program (Textfile S1) that generates homology, identity and blosum scores on the basis of Blosum62 matrix (Dayhoff *et al*., 1978), modified to account for loops (score −5), missing amino acids (0) and gaps (0) (Textfile S2).

### Construction of vectors

Primers and restriction sites are in Table S3. The integration vector, pIRCTGFP, was generated by inserting ∼1.3 kb of calpain 3′ end from 3D7 genome into pPM2GT (Klemba *et al*., 2004). Complementary oligonucleotides encoding 6-his, 2×-myc or 2×-flag tags, flanked by AvrII/NotI cohesive ends (Table S3), were annealed and phosphorylated with T4 kinase.

Plasmids for episomal expression of the YFP chimeras were constructed as follows. First, sequences encoding calpain fragments were produced from 3D7 DNA and cloned into peYFP-N1 (Clontech) (Table S3). This vector was used for mammalian transfection and for subcloning into *Plasmodium* vectors. For *Plasmodium* episomal expression, the Hsp86-5′UTR and the Rep20 element were inserted into pIRCTGFP. In this backbone we cloned all the YFP-chimeras, as well as YFP alone. For the multi-YFP construct, an amplified YFP fragment was digested with Acc65I and BsrGI and cloned with orientation screening in BsrGI-digested peYFP-N1. td-Tomato was amplified from pRSET-td-Tomato (Shaner *et al*., 2004).

### Radioactive labelling, cell extracts, immunoblotting and immunoprecipitation

For radioactive labelling, 293T cells were incubated overnight with 250 μCi ^3^H-myristic or ^3^H-palmitic acid (Perkin-Elmer, 30 Ci mmol^−1^ each) in media with 0.5% serum. Monolayers were washed with cold PBS and lysed in RIPA buffer (50 mM Tris-HCl pH 8.0, 150 mM NaCl, 1% NP40, 0.5% deoxycholate, 0.1% SDS) containing 5 mM EDTA and complete protease inhibitors (PI, Roche). Samples were either immunoprecipitated or electrophoretically analysed.

Parasites from one to three plates of asynchronous parasites at high parasitemia, 2% hematocrit were collected after tetanolysin treatment (25 units per plate, 5–10 min at room temperature) in PBS and lysed in RIPA buffer. Buffers contained PI, 5 mM EDTA and 10 μM pepstatin.

Immunoprecipitation from parasites or 293T cells expressing short-calpain chimeras was performed with 5 μl of packed protein-G dynabeads (Invitrogen) conjugated according to manufacturer protocol with rabbit anti-GFP (1 μl per 20 μl beads) or mouse anti-GFP (1 μl per 5 μl beads).

To immunoprecipitate full-length calpain, the 1% SDS-boiled lysate was diluted 20 times in RIPA buffer with PI, pepstatin, 5 mM MgSO_4_, 1 U DNAse and 0.5 μg RNAse. Ten microlitres of anti-calpain #19 or 5 μl of rabbit anti-GFP was added, incubated overnight at 4°C and pulled down with 40 μl of protein A-agarose (Amersham).

Immune complexes were boiled in reducing or non-reducing (for palmitoylation detection) SDS-PAGE sample buffer. Full-length calpain was fractionated on 3–8% NuPAGE (Invitrogen) with SDS-Tris-acetate buffer according to manufacturer instructions. Gels were transferred to nitrocellulose in presence of 0.1% SDS. For immunodetection we used 5% milk as blocking reagent.

For cellular fractionation tetanolysin-isolated parasites were lysed by sonication in PBS containing PI, EDTA and pepstatin as above. A centrifugation at 10 000 *g* for 10 min was performed to remove cellular debris and then soluble and membrane fractions were obtained by ultracentrifugation at 100 000 *g* for 30 min at 4°C. Membranes were washed once with the same buffer, using sonication for resuspension. Samples were analysed by WB.

### Southern blots

For Southern blots, 1 μg of each sample DNA was digested overnight and processed (Klemba *et al*., 2004). The C-terminal ORF integrations were screened by NsiI/SphI digestion and probed with DNA corresponding to the C-terminus.

### Microscopy techniques

Live parasites were observed in presence of Hoechst33342 or SytoRNA (Molecular Probes). Images were collected with an Axioskop epifluorescence microscope (Klemba et al., 2004).

Parasites were fixed, permeabilized (Ponpuak *et al*., 2007) and incubated with antibody in a humidified chamber. Cells were pre-layered on PEI-treated coverslips and mounted in DAPI-containing Vectashield (Vector Laboratories) or Prolong Gold antifade (MolecularProbes). Nuclei were isolated (Lanzer *et al*., 1992), air-dried on a glass slide and quickly methanol-fixed. The secondary antibodies were Alexafluor 488- or 555-conjugated (MolecularProbes).

Prior to microscopy analysis 293T cells were grown on gelatin-pretreated coverslips. Confocal images were acquired with a LSM 510 Meta laser scanning confocal microscope (Carl Zeiss) equipped with a 63×, 1.4 n.a. Zeiss Plan Apochromat oil objective.

For EM, parasitized RBC were fixed as described above, treated with 25 U of tetanolysin for 30 min at room temperature, washed and incubated with primary antibody followed by colloidal gold conjugated anti-rabbit (12 nm) and anti-mouse (18 nm) IgG (Jack Imm Res Laboratory) before processing for EM as described (Ponpuak *et al*., 2007).

### Reagents and antibodies

All reagents were purchased from Sigma or NEB unless indicated. Polyclonal antibodies against Pf_calpain were raised in rabbits using calpain peptides: 3–22 (serum #17); 41–54 (#19); 1805–1821 (#35) (Fig. S1). Other antibodies: rabbit anti-GFP 6556 (Abcam) diluted for microscopy (Mic) 1:200 and for WBs 1:5000; goat anti-GFP 5450 (Abcam) Mic 1:25; monoclonal anti-GFP (JL8, BD) WB 1:1000; rabbit anti-calpain #17 WB 1:250; monoclonal Anti-flag M2 Mic 1:100; monoclonal anti-His Mic 1:100; rat and rabbit anti-Bip (MR4) Mic 1:1500 and WB 1:10 000; monoclonal anti-hNop1 (Abcam) Mic 1:12.5; polyclonal anti-Pf_Nop1 (Figueiredo *et al*., 2005); Mic 1:25; anti-calpain #35 Mic 1:50 (whole cell) and 1:400 (purified nuclei); monoclonal anti-PMV (Klemba and Goldberg, 2005), WB 1/400.

Two secondary antibodies were used, anti-mouse and anti-rabbit, both HRP-conjugated (Amersham).
