# Supplementary material for: Fatty acid acylation regulates trafficking of the unusual Plasmodium falciparum calpain to the nucleolus
Source: Mol Microbiol. 2009 Mar 2;72(1):229–45. doi: 10.1111/j.1365-2958.2009.06639.x (PMC2746569; doi:10.1111/j.1365-2958.2009.06639.x)
Supplement: Supplementary file 1 [file mmi0072-0229-SD1.pdf]

Supporting Information

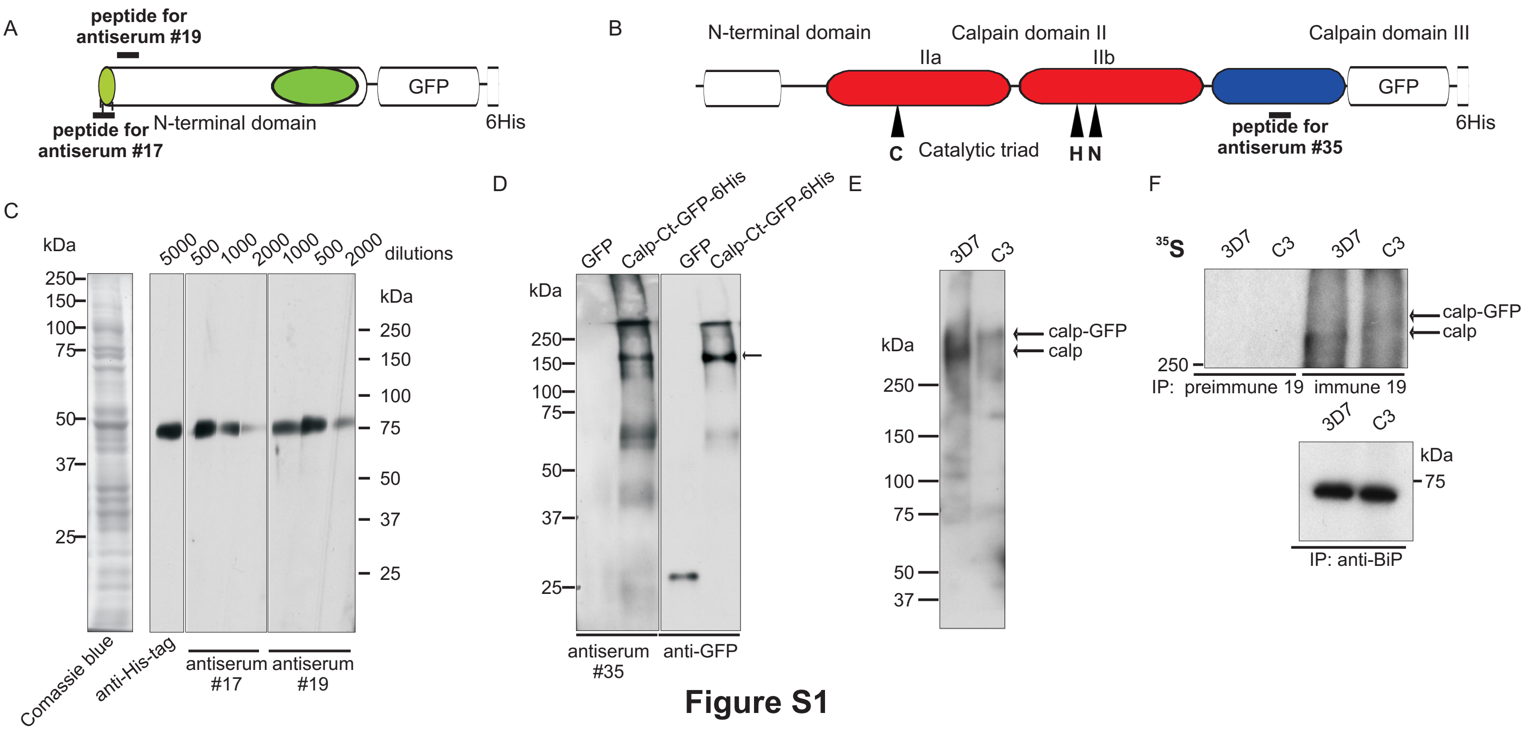

Fig. S1. Validation of anti-calpain antisera used in the present work.

Schematics illustrating the Pf_calpain fragments, N-terminal 421aa (A) and C-terminal 1273aa (B) fused to a GFP-6His tag, expressed using the wheat germ in vitro system (Cellfree science) according to the manufacturer's protocol. Locations of synthetic peptides used to raise rabbit anti-calpain antisera are shown as black bars. (C) Western blot of total protein extract expressing the N-terminus. Antibodies and dilutions are indicated: mouse-anti-his (sigma), rabbit anti-calpain #17 antiserum and rabbit anti-calpain #19 antiserum. Panels are discontiguous areas of the same membrane. Coomassie staining of the extract is shown in the left panel. (D) Western blot of total protein extract expressing the calpain C-terminus or GFP. Antibodies and dilutions are shown: mouse-anti-GFP (Sigma), rabbit anti-calpain #35. The proteins expressed by the wheat germ system were GFP (lanes 1 and 3) and Calp-Ct-GFP-His (lanes 2 and 4). (E) Western blot analysis of protein extracts derived from asynchronous 3D7 parental parasites and Calpain-GFP expressing parasites (C3). Each lane corresponds to ~1.2 x10^8 parasites, separated by Tris-acetate PAGE (3-8%), and transferred in the presence of 0.1% SDS. The primary antibody was rabbit anti-calpain #19 antiserum, the detection was conducted using HRP-conjugated anti-rabbit antibody and ECL+ solutions (Amersham). The exposure time was over 30 min. F. Immunoprecipitation of Pf_calpain from ^{35}S-labelled parasites using rabbit anti-calpain #19 antiserum and its preimmune serum. Parasites were labeled for 24h using (EXPRE^{35}S^{35}S, NEN) as previously described (Klemba, M. *et al.*, *J Cell Biol,* 2004, 164, pp47-56). From the same experiment BiP has been immunoprecipitated as a loading control (bottom panel).

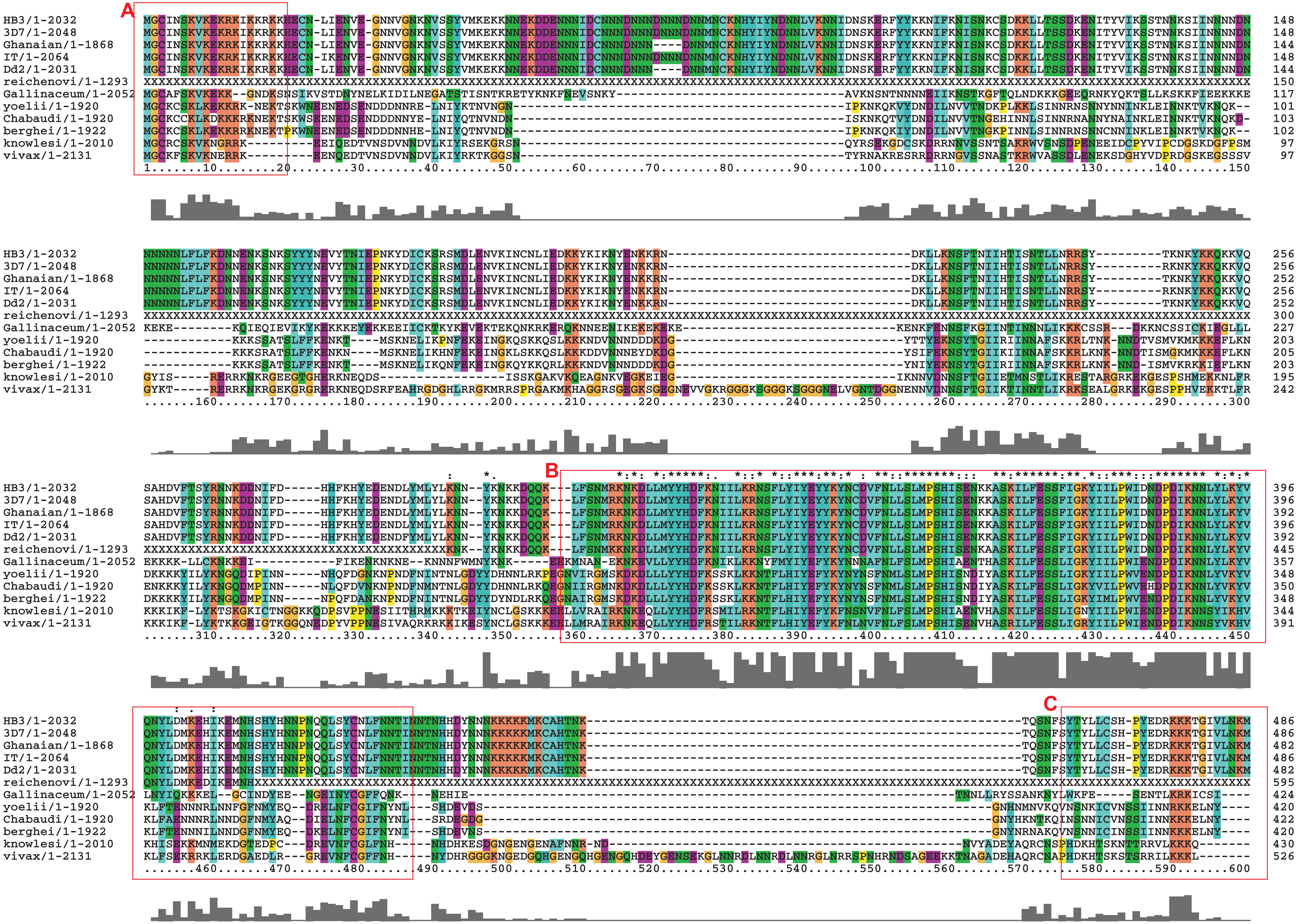
A
B
C
HB3/1-2032
3D7/1-2048
Ghanaian/1-1868
IT/1-2064
Dd2/1-2031
reichenovi/1-1293
Gallinaceum/1-2052
yoelii/1-1920
Chabaudi/1-1920
berghei/1-1922
knowlesi/1-2010
vivax/1-2131

Figure S2

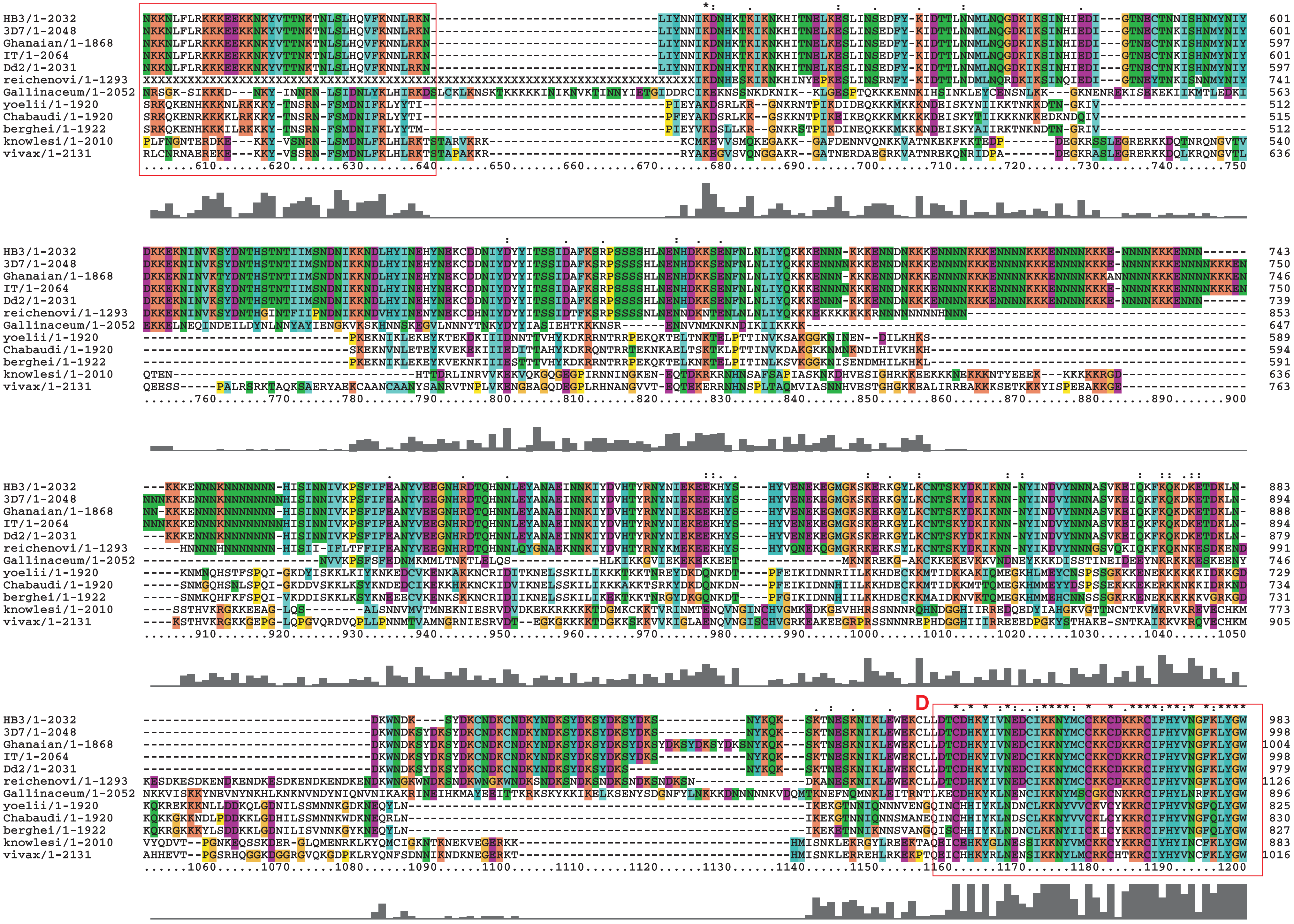
HB3/1-2032
3D7/1-2048
Ghanaian/1-1868
IT/1-2064
Dd2/1-2031
reichenovi/1-1293
Gallinaceum/1-2052
yoelii/1-1920
Chabaudi/1-1920
berghei/1-1922
knowlesi/1-2010
vivax/1-2131
601
601
597
601
597
741
563
512
515
512
540
636
743
750
746
750
739
853
647
589
594
591
636
763
883
894
888
894
879
991
746
729
734
731
773
905
D
983
998
1004
998
979
1126
896
825
830
827
883
1016

Figure S2

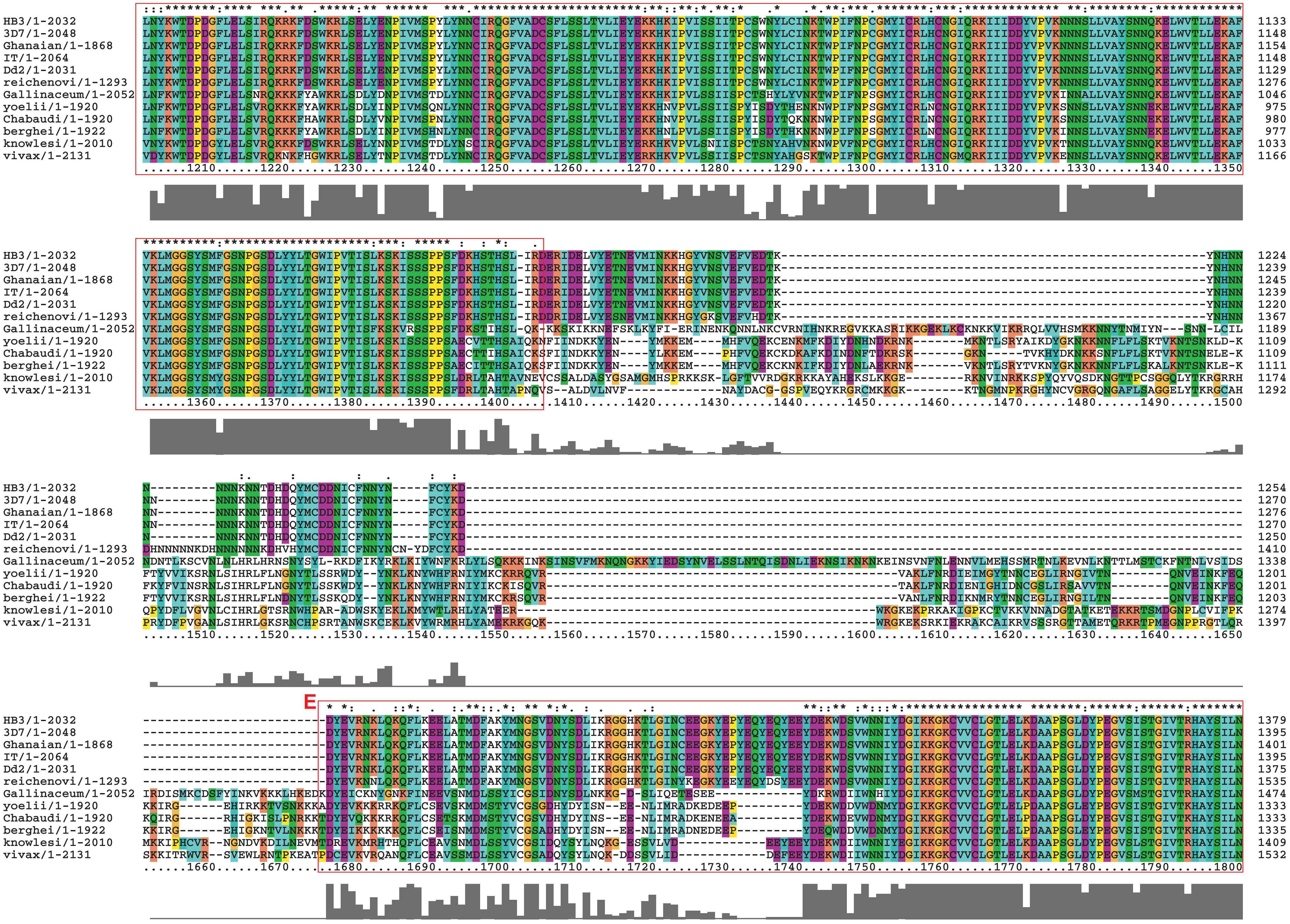
HB3/1-2032
3D7/1-2048
Ghanaian/1-1868
IT/1-2064
Dd2/1-2031
reichenovi/1-1293
Gallinaceum/1-2052
yoelii/1-1920
Chabaudi/1-1920
berghei/1-1922
knowlesi/1-2010
vivax/1-2131
E

Figure S2

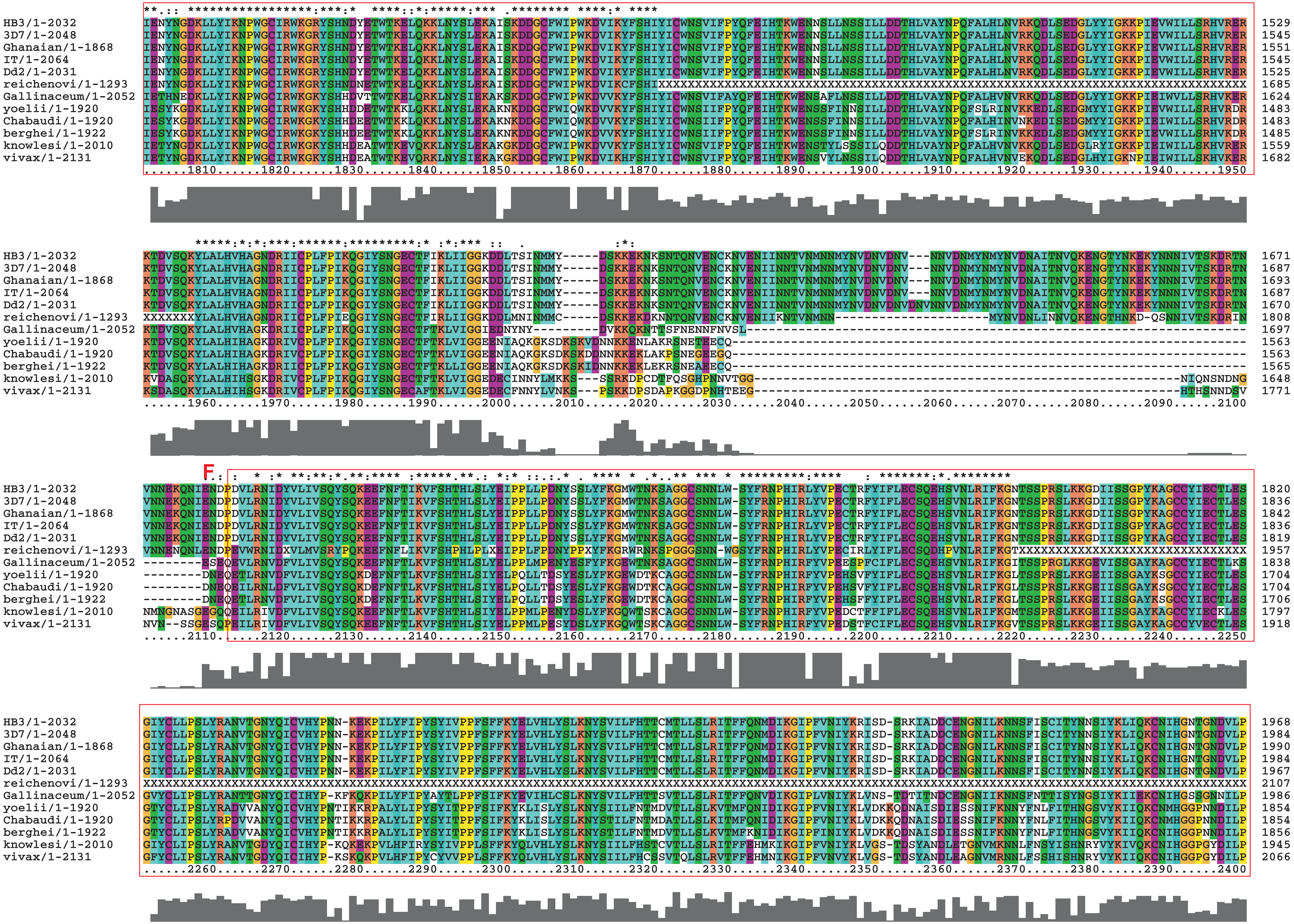

Figure S2

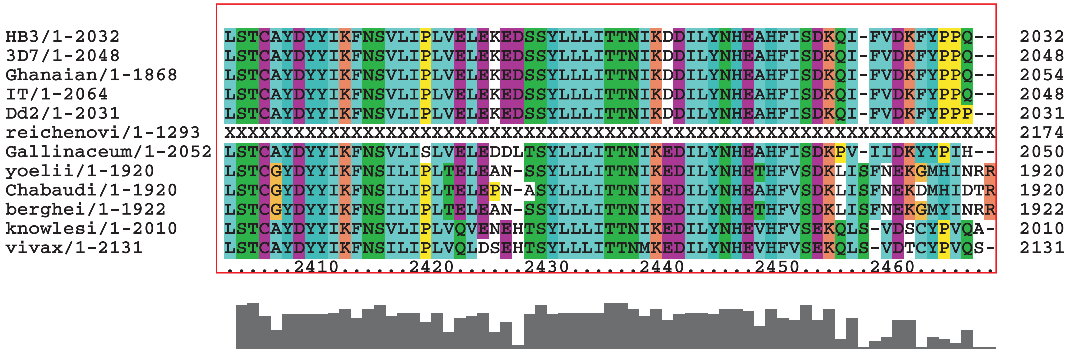

Fig. S2. Amino acid alignment of *Plasmodium* calpains.

All available *Plasmodium* calpains, listed in Table S1, were aligned using ClustalX and Jalview. Quality of alignment is shown below the sequences. HC1 (a), HC2 (b), HC3 (c), HC4 (d), HC5 (e), HC6 (f) are shown as boxes. Red arrows indicate the catalytic triad position.

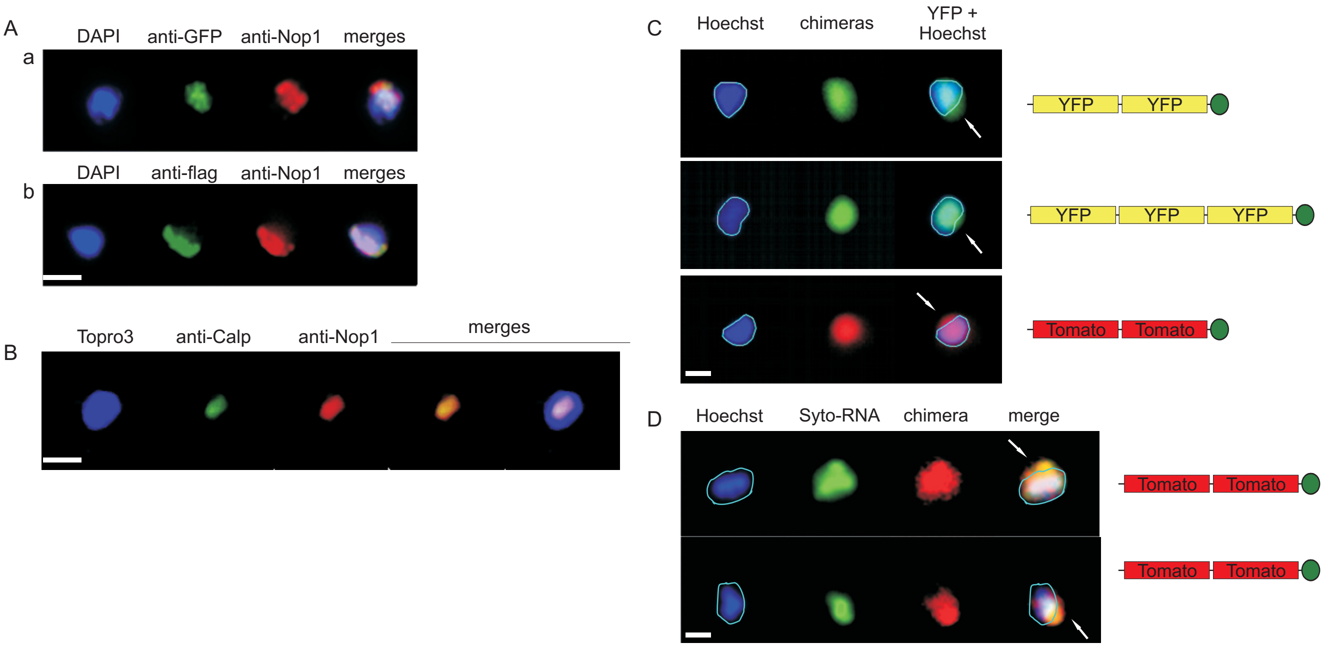

Figure S3

Fig. S3. Enlargements of parasite nuclei.

(A) Co-localization of Nop1 and calpain-GFP (a) or calpain-flag (b), from Fig. 3A. (B) Co-localization of calpain and Nop1 in a purified 3D7 nucleus. A confocal image of sample immunostained with antibodies specific for Pf_Calpain (#35, rabbit) and hNop1 (mouse) is shown. The nucleus was stained with Topro3. (C and D) Enlarged nuclei of parasites expressing the small chimeras presented in Fig. 5, panels D (panel C) and H (panel D). On the right of the panels are schematics of the constructs expressed. Arrows indicate the position of the nucleolus that in live cells appears clearly negative for the DNA staining. The nuclei are outlined. In D nuclei

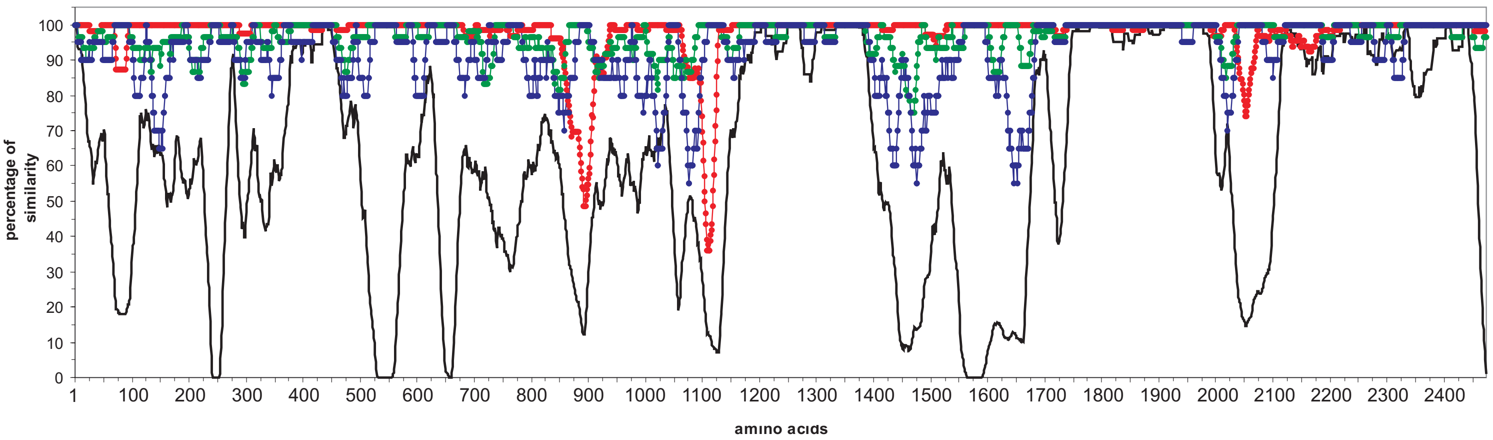

Figure S4

Fig. S4. Calpain conservation in *Plasmodium* species.

In the graph, the x-axis shows amino acid position along the alignment shown in Fig. S2. The graph shows the percentages of similarity among calpains encoded by *Plasmodium* species (black line) (*P. falciparum*, *reichenowi*, *gallinaceum*, *yoelii yoelii*, *berghei*, *chabaudi*, *knowlesi* and *vivax* (Table S1)); by *P. falciparum* and *reichenowi* (red); *P. yoelii yoelii*, *berghei* and *chabaudi* (green); *P. knowlesi* and *vivax* (blue). The conservation degree was calculated in a 20 amino acid sliding window throughout the aligned calpain sequences (PERL program in Textfile S1).

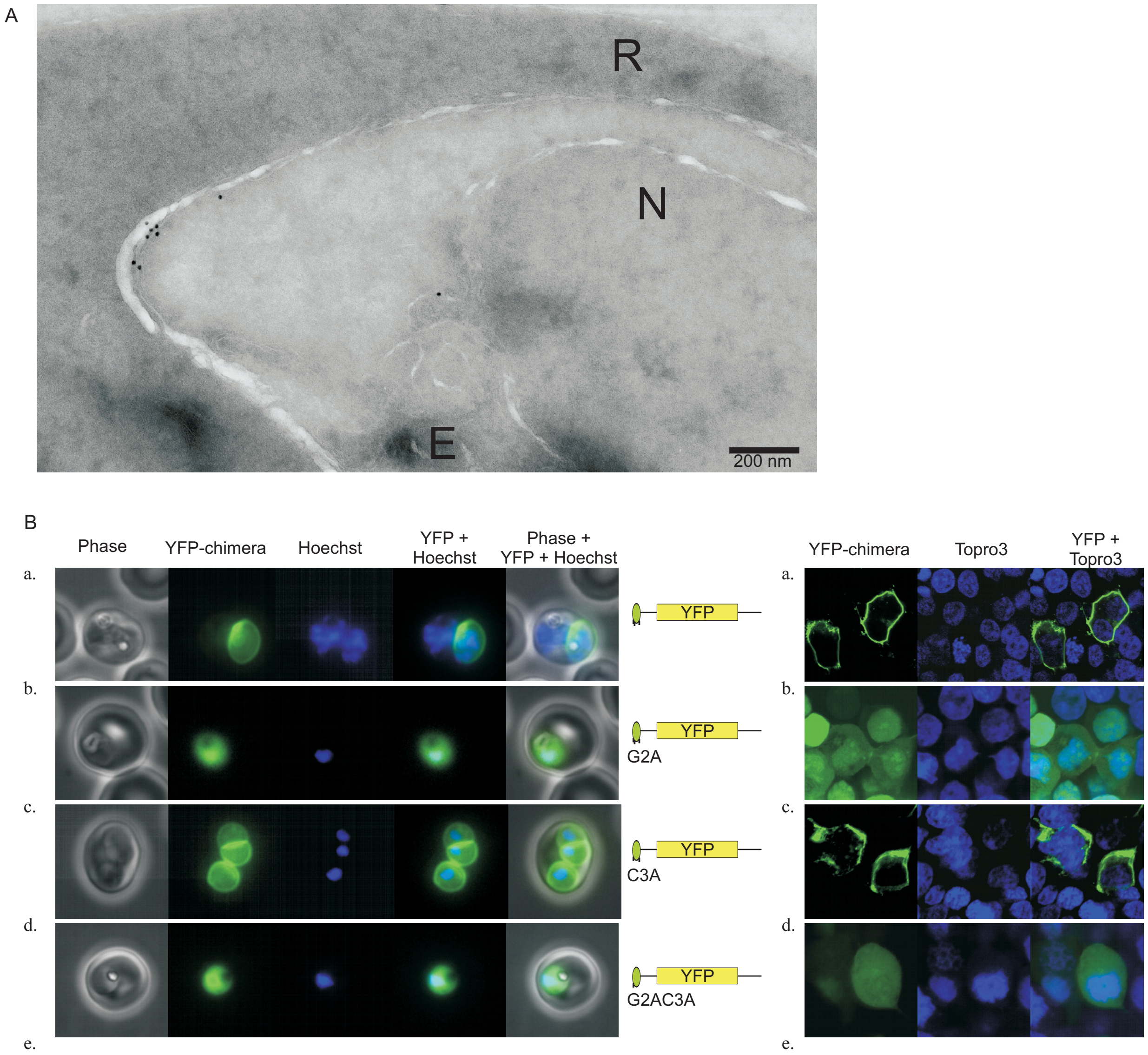

Figure S5

Fig. S5. Localization of Pf_calpain NtL-YFP chimeras.

(A) Immuno-EM of *P. falciparum* expressing HC1-YFP chimera. The image presents a trophozoite section where a parasite (nucleus (N) and endoplasmic reticulum (E)) is visible enclosed in a red blood cell (R). At the periphery, on the PM, significant labeling is due to the immunodetection of YFP (gold particles 12 nm). (B) Fluorescence profile of live transfected *P. falciparum* (left) and fixed human 293T cells (right) expressing NtL. Between the two panels are schematics of the constructs expressed. Light green oval, HC1 subdomain. Below each schematic is the mutation constructed. Images are labeled at the top.

Table S1. *Plasmodium* strains and calpain ORFs used in this work

	sequence source	contigs, fragments or gene loci	HC1 (a.a.)	PC1 (a.a.)	HC2 (a.a.)	PC2 (a.a.)	HC3 (a.a.)	PC3 (a.a.)	HC4 (a.a.)	PC4 (a.a.)	HC5 (a.a.)	PC5 (a.a.)	HC6 (a.a.)	synonymous mutations due to SNPs*	non-synonymous due to SNPs*
***P. falciparum* HB3**	Broad institute	supercontig 55 and present work published in Genbank432833	31	273	126	36	59	417	239	135	280	80	353	0	1 (in PC3)
***P. falciparum* 3D7**	GeneDB	MAL13P1.310 and present work published in Genbank432831-2	31	273	126	36	59	432	242	136	280	80	353	0	1 (in PC1)
***P. falciparum* Ghanian**	Sanger institute	(clinic isolated) Contig20779; pfclin1989d07.q1k; pfclin151b06.p1k; pfclin1195f02.p1k	31	269	126	36	59	442	239	136	280	80	353	0	3 (in PC1, PC3)
***P. falciparum* IT**	Sanger institute	it315b05.q1k, it132f09.p1k	32	273	126	36	59	432	239	136	280	80	353	0	3 (in PC1)
***P. falciparum* Dd2**	Broad institute	supercontig 352	31	269	126	36	59	417	239	135	280	83	353	2 (in HC6)	3 (in PC1, PC3, HC6***)
P. reichenowi	Sanger institute	reich418g07.p1k, reich792d12.p1k, reich418g07.q1k, reich1116d08.p1k, reich801d11.p1k, reich1116d08.q1k, reich801c06.q1k, reich801d11.q1k, reich309f08.q1k	-	12**	107**	-	-	424**	239	148	194**	49	105**	-	-
P. gallinaceum	Sanger institute	gal28a.d000000717.Contig1	33	231	123	18	67	360	239	322	280	7	353	-	-
***P. yoelii yoelii* 17XNL**	PlasmoDB	PY00976	32	225	125	23	53	328	239	247	285	5	356	-	-
P. chabaudi chabaudi	Sanger institute	PC000118.05.0 and PC000283.01.0	33	226	125	23	53	331	239	252	285	5	356	-	-
***P. berghei* ANKA**	PlasmoDB	PB001043.00.0	33	225	125	23	53	330	239	247	285	5	356	-	-
P. knowlesi	Sanger institute	Pk_20g02p1c and Pk_275g10p1c	22	229	124	32	65	370	239	265	282	25	354	-	-
***P. vivax* Sal-I**	Sanger institute	Pv115075	22	276	124	81	65	457	239	252	282	23	354	-	-

* These values were derived from the analysis of *P. falciparum* species only using 3D7 sequence as reference.

** These numbers do not indicate the actual length of the domain because they are derived from uncompleted ORF sequencing.

*** This SNP in HC6 changes the very last amino acid of the sequence

Table S2. Proteins with a motif homologous to HC2.

Hit	score	method
NP_705040.1- hypothetical protein *P.f.* 3D7	45.2	Short and exact match
NP_704561.1- hypothetical protein *P.f.* 3D7	41.8	Short and exact match
XP_679118.1 - 26S proteasome reg. subunit S14 *P.b.* ANKA	40.1	Protein-protein
NP_702393.1 - hypothetical protein PF14_0504 *P.f.* 3D7	38.8	Protein-protein
XP_317018.2 - ENSANGP00000019200 *An. gambiae*	37.1	Protein-protein
NP_702312.1 - putative Ser/Thr protein kinase *P.f.* 3D7	36.7	Short and exact match
XP_731318.1 – cys repeat modular protein *P. y.* 17XNL	36.7	Protein-protein
NP_701275.1 - putative myosin heavy chain subunit *P.f.* 3D7	36.3	Short and exact match
NP_704042.1 - hypothetical protein *P.f.* 3D7	35.8	Protein-protein
NP_705383.1 - putative ATP binding protein *P.f.* 3D7	35.8	Protein-protein
CAA64576.1 - rps4 *P.f.* 3D7	35.8	Protein-protein
XP_628012.1 - tRNA (Gm18) ribose methylase; trm3p; SpoU superfamily *Cryptosp. parvum* Iowa II	35.4	Protein-protein
CAA64574.1 – rpoD *P.f.* 3D7	35	Protein-protein
NP_764804.1 - DNA primase *Staph. epidermidis*	34.6	Protein-protein
XP_568548.1 - nucleolus prot. *Cryptoc. Neoformans* JEC21	34.2	Protein-protein
YP_270218.1 - transcriptional activator *Colw. psych.*	34.1	Protein-protein
EAR86119.1 – Adenylate/Guanylate cyclase catalytic dom. *Tetrah. therm.*	33.7	Protein-protein
XP_728972.1 - rRNA methylase *P. y.* 17XNL	33.7	Protein-protein
YP_661775.1 - transcriptional regulator *Ps. atlantica*	33.3	Protein-protein
Various hits similar to N-acetyltransferase-like prot.	33.9	Protein-protein
Various hits similar to guanine nucleotide-binding prot.	32.5	Short and exact match
ABE87709 - DEAD/DEAH box helicase, N-term. *M. tr.*	32	Protein-protein
NP_700706.1 - hypothetical protein PF10_0233 *P.f.* 3D7	25	Protein-protein
NP_704458.1 - hypothetical protein *P.f.* 3D7	24.6	Protein-protein
XP_966228.1 - hypothetical protein PFF1185w *P.f.* 3D7	24.6	Protein-protein
NP_702369.1 - hypothetical protein PF14_0480 *P.f.* 3D7	23.9	Protein-protein

Table S3. Primers used in this work.

#	Primer	Sequence	Orientation	Restriction site	Purpose	comments
IR10	5'flXhoI	TACATTCTCGAGGTTGATAATAGGTGGTAAGG	Forward	XhoI	Ct-PCR	
IR11	5'flAvrII	TAAAAGCCTAGGTTGGGGTGGATAAAATTTATC C	Reverse	AvrII	Ct-PCR	
IR42	prehisfor	CTAGGCTGGAAGTTCTGTTCCAGGGGCCCCATC ATCATCATCATCATCATCATTAAC	Forward	AvrII sticky end	PreScission-Histag	
IR43	prehisrev	GGCCGTTAATGATGATGATGATGATGATGATGG GGCCCCTGGAACAGAACTTCCAGC	Reverse	EagI sticky end	PreScission-Histag	
IR46	preflagfor	CTAGGCTGGAAGTTCTGTTCCAGGGGCCCGATT ACAAGGATGACGACGATAAGGATTACAAGGATG ACGACGATAAGTAAGC	Forward	AvrII sticky end	PreScission-Flagtag	
IR47	preflagrev	GGCCGCTTACTTATCGTCGTCATCCTTGTAATC CTTATCGTCGTCATCCTTGTAATCGGGCCCCTG GAACAGAACTTCCAGC	Reverse	EagI sticky end	PreScission-Flagtag	
IR50	premycfor	CTAGGCTGGAAGTTCTGTTCCAGGGGCCCGAG CAGAAACTCATCTCTGAAGAGGATCTGGAGTAA C	Forward	AvrII sticky end	PreScission-Myctag	
IR51	premycrev	GGCCGTTACTCCAGATCCTCTTCAGAGATGAGT TTCTGCTCGGGCCCCTGGAACAGAACTTCCAGC	Reverse	EagI sticky end	PreScission-Myctag	
IR1wb	Ntfor	TCGAGATGGGGTGTATAAATAGCAAAGTAAAAG AGAAAAGAAAAATAAAAAAAAGAAAAAAGGAAGA ATGTAATTTACG	Forward	XhoI sticky end	Calpain Nt (1-24)	
IR2wb	Ntrev	AATTCGTAAATTACATTCTTCCTTTTTTCTTTTTT TTATTTTTCTTTTCTCTTTTACTTTGCTATTTATA CACCCCATC	Reverse	EcoRI sticky end	Calpain Nt (1-24)	
IR3wb	Ntlongfor	TCGAGATGGGGTGTATAAATAGCAAAGTAAAAG AGAAAAGAAAAATAAAAAAAAGAAAAAAGGAAGA ATGTAATTTAATAGAAAATGTTGAAGGGAATAAT GTGGGTCG	Forward	XhoI sticky end	Calpain Nt (1-34)	
IR4wb	Ntlongrev	AATTCGACCCACATTATTCCCTTCAACATTTTCT ATTAAATTACATTCTTCCTTTTTTCTTTTTTTTATT TTTCTTTTCTCTTTTACTTTGCTATTTATACACCC CATC	Reverse	EcoRI sticky end	Calpain Nt (1-34)	
IR5wb	5'nls	ATGTAGTCTGTACAGGGAAGATCGAAAAAAAAA AACAGGC	Forward	BsrGI	Calpain NLS PCR	

IR6wb	3'nls	GAAGACTAAGGCGGCCGCTTTAAGTAGTAACAT ATTTATTTTTCTTTTCTTC	Reverse	NotI	Calpain NLS PCR
IR200	G2A	CCGCTAGCGCTACCGGACTCAGATCTCGAGATG GCGTGTATAAATAGCAAAGTAAAAGAG	Forward	-	Mutagenesis G2A
IR201	G2Arev	CTCTTTTACTTTGCTATTTATACACGCCATCTCG AGATCTGAGTCCGGTAGCGCTAGCGG	Reverse	-	Mutagenesis G2A
IR202	C3A	CCGCTAGCGCTACCGGACTCAGATCTCGAGATG GGGGCTATAAATAGCAAAGTAAAAGAG	Forward	-	Mutagenesis C3A
IR203	C3Arev	CTCTTTTACTTTGCTATTTATAGCCCCCATCTCG AGATCTGAGTCCGGTAGCGCTAGCGG	Reverse	-	Mutagenesis C3A
IR204	G2AC3A	CCGCTAGCGCTACCGGACTCAGATCTCGAGATG GCGGCTATAAATAGCAAAGTAAAAGAG	Forward	-	Mutagenesis G2AC3A
IR205	G2AC3Arev	CTCTTTTACTTTGCTATTTATAGCCGCCATCTCG AGATCTGAGTCCGGTAGCGCTAGCGG	Reverse	-	Mutagenesis G2AC3A
IR204	C22A	GAAAAATAAAAAAAAGAAAAAAGGAAGAAGCTAA TTTAATAGAAAATGTTGAAGGG	Forward	-	Mutagenesis C22A
IR205	C22Arev	CCCTTCAACATTTTCTATTAAATTAGCTTCTTCCT TTTTTCTTTTTTTTATTTTTC	Reverse	-	Mutagenesis C22A
IR267	multi-YFPforw	CAGTCGACGGTACCGGGCCCGGGATCCACCGG TCGCCACCATGGTGAGCAAGGGCGAGGAGC	Forward	Acc65I	multi-YFP

```
##################################################################################
##################################################################################
##Program to evaluate similarity, identity and blosum score                     ##
##in a sliding Window along an alignment                                        ##
##                                                                              ##
##Co-Authors:                                                                   ##
##Michael D. Brooks(1) & Ilaria Russo(2)                                        ##
##                                                                              ##
##(1)Genome Sequencing Center                                                   ##
##Washington University School of Medicine                                      ##
##St. Louis, MO, USA - mdbrooks@artsci.wustl.edu                                ##
##                                                                              ##
##(2)Departments of Medicine and Molecular Microbiology                         ##
##Howard Hughes Medical Institute                                               ##
##Washington University School of Medicine,                                     ##
##St. Louis, MO, USA - russo@borcim.wustl.edu                                   ##
##                                                                              ##
##################################################################################
##################################################################################


############################################
## sliding basepair Window Size           ##

my $N = 20;

##                                        ##
############################################


############################################
## number of sequences in the alignment   ##

my @first_seq_array = ();
my @second_seq_array = ();
my @third_seq_array = ();
my @fourth_seq_array = ();
my @fifth_seq_array = ();
my @sixth_seq_array = ();
my @seventh_seq_array = ();
my @eighth_seq_array = ();
my @ninth_seq_array = ();
my @tenth_seq_array = ();
my @eleventh_seq_array = ();
my @twelfth_seq_array = ();
my $first_sequence = ();
my $second_sequence = ();
my $third_sequence = ();
```

```
my $fourth_sequence = ();
my $fifth_sequence = ();
my $sixth_sequence = ();
my $seventh_sequence = ();
my $eighth_sequence = ();
my $ninth_sequence = ();
my $tenth_sequence = ();
my $eleventh_sequence = ();
my $twelfth_sequence = ();

##                                        ##
############################################


###########################################################
## code for each amino acid, gap (-) and missing aa (X)  ##

my $line;

my %amino_acid_code = (
 'A' => '0',
 'R' => '1',
 'N' => '2',
 'D' => '3',
 'C' => '4',
 'Q' => '5',
 'E' => '6',
 'G' => '7',
 'H' => '8',
 'I' => '9',
 'L' => '10',
 'K' => '11',
 'M' => '12',
 'F' => '13',
 'P' => '14',
 'S' => '15',
 'T' => '16',
 'W' => '17',
 'Y' => '18',
 'V' => '19',
 '-' => '20',
 'X' => '21',
);

##                                                      ##
##########################################################
```

```
###############################################################################################
## Acquire the blosum matrix values                                                          ##

open (IN, shift @ARGV);
my @blosum_array = ();
my $loop_counter = 0;
while ($line = <IN>){
        my @temp_line_array = split(" ", $line);
        my $temp_counter = scalar @temp_line_array;
        for (my $i = 0; $i < $temp_counter; $i++){
                $blosum_array[$loop_counter][$i] = shift @temp_line_array;
        }
        $loop_counter++;
}
close IN;
##
##                                                                                           ##
###############################################################################################


###########################################################
## Assign a variable to each sequence of the alignment   ##
## If variable equals 1, the sequence will be analyzed;  ##
## if 0, it won't                                        ##

my $aa = 1;
my $bb = 1;
my $cc = 1;
my $dd = 1;
my $ee = 1;
my $ff = 1;
my $gg = 1;
my $hh = 1;
my $ll = 1;
my $mm = 1;
my $nn = 1;
my $oo = 1;

##                                                       ##
###########################################################


###############################################################################################
## open the alignment and create a matrix for each sequence in it                            ##

open (IN, shift @ARGV);
while($line = <IN>){
        if ($aa == 1){
```

```
		if ($line =~ /^	#"name of the first seq"#	/){
			my @line_array = split(" ", $line);
			my $sequence = pop @line_array;
			$first_sequence = $first_sequence.$sequence;
			@first_seq_array = split("", $first_sequence);
		}
	}
	if ($bb == 1){
		if ($line =~ /^	#"name of the second seq"#	/){
			my @line_array = split(" ", $line);
			my $sequence = pop @line_array;
			$second_sequence = $second_sequence.$sequence;
			@second_seq_array = split("", $second_sequence);
		}
	}
	if ($cc == 1){
		if ($line =~ /^	#"name of the third seq"#	/){
			my @line_array = split(" ", $line);
			my $sequence = pop @line_array;
			$third_sequence = $third_sequence.$sequence;
			@third_seq_array = split("", $third_sequence);
		}
	}
	if ($dd == 1){
		if ($line =~ /^	#"name of the fourth seq"#	/){
			my @line_array = split(" ", $line);
			my $sequence = pop @line_array;
			$fourth_sequence = $fourth_sequence.$sequence;
			@fourth_seq_array = split("", $fourth_sequence);
		}
	}
	if ($ee == 1){
		if ($line =~ /^	#"name of the fifth seq"#	/){
			my @line_array = split(" ", $line);
			my $sequence = pop @line_array;
			$fifth_sequence = $fifth_sequence.$sequence;
			@fifth_seq_array = split("", $fifth_sequence);
		}
	}
	if ($ff == 1){
		if ($line =~ /^	#"name of the sixth seq"#	/){
			my @line_array = split(" ", $line);
			my $sequence = pop @line_array;
			$sixth_sequence = $sixth_sequence.$sequence;
			@sixth_seq_array = split("", $sixth_sequence);
		}
	}
	if ($gg == 1){
```

```
        if ($line =~ /^      #"name of the seventh seq"#     /){
                my @line_array = split(" ", $line);
                my $sequence = pop @line_array;
                $seventh_sequence = $seventh_sequence.$sequence;
                @seventh_seq_array = split("", $seventh_sequence);
        }
}
if ($hh == 1){
        if ($line =~ /^      #"name of the eighth seq"#     /){
                my @line_array = split(" ", $line);
                my $sequence = pop @line_array;
                $eighth_sequence = $eighth_sequence.$sequence;
                @eighth_seq_array = split("", $eighth_sequence);
        }
}
if ($ll == 1){
        if ($line =~ /^      #"name of the ninth seq"#     /){
                my @line_array = split(" ", $line);
                my $sequence = pop @line_array;
                $ninth_sequence = $ninth_sequence.$sequence;
                @ninth_seq_array = split("", $ninth_sequence);
        }
}
if ($mm == 1){
        if ($line =~ /^      #"name of the tenth seq"#     /){
                my @line_array = split(" ", $line);
                my $sequence = pop @line_array;
                $tenth_sequence = $tenth_sequence.$sequence;
                @tenth_seq_array = split("", $tenth_sequence);
        }
}
if ($nn == 1){
        if ($line =~ /^      #"name of the eleventh seq"#     /){
                my @line_array = split(" ", $line);
                my $sequence = pop @line_array;
                $eleventh_sequence = $eleventh_sequence.$sequence;
                @eleventh_seq_array = split("", $eleventh_sequence);
        }
}
if ($OO == 1){
        if ($line =~ /^      #"name of the twelfth seq"#     /){
                my @line_array = split(" ", $line);
                my $sequence = pop @line_array;
                $twelve_sequence = $twelve_sequence.$sequence;
                @twelve_seq_array = split("", $twelve_sequence);
        }
}
```

```
}

close IN;

##                                                                                            ##
################################################################################################


################################################################################################
##For each single position in the alignment a matrix is created containing blosum             ##
##scores for each amino acid-amino acid comparison                                            ##

my @score_array = ();
my @similarity_array = ();
my @identity_array = ();

my $counter = scalar @first_seq_array + 1;
print "counter is $counter\n";

for(my $i = 0; $i < $counter; $i++){
        my $final_score = 0;
        my $score = 0;
        my $similarity = 0;
        my $similarity_a = 0;
        my $similarity_aa = 0;
        my $seqq = 0;
        my $seqq1 = 0;
        my $identity_counter = 0;
        my $identity = 0;
        my $identity1 = 0;
        my $identity2 = 0;
        $seqq = $aa + $bb + $cc + $dd + $ee + $ff + $gg + $hh + $ll + $mm + $nn + $oo;
        $seqq1 = $seqq;


        my @first_aa_array = ($first_seq_array[$i], $second_seq_array[$i], $third_seq_array[$i],
                                $fourth_seq_array[$i], $fifth_seq_array[$i], $sixth_seq_array[$i],
                                $seventh_seq_array[$i], $eighth_seq_array[$i], $ninth_seq_array[$i],
                                $tenth_seq_array[$i], $eleventh_seq_array[$i], $twelfth_seq_array[$i]);
        my @second_aa_array = @first_aa_array;

        ################################################################################################
        ##For each conservative blosum score (>0) the similarity score is                             ##
        ##increased by a unit, excluding the self comparison; each missing amino acid is              ##
        ##removed from the analysis; and all loops/gaps are not scored as conserved position          ##

        for (my $j = 0; $j <$seqq; $j++){
```

```
		for (my $k = 0; $k < $seqq; $k++){
			my $first_aa = $first_aa_array[$j];
			my $second_aa = $second_aa_array[$k];
			my $first_num = $amino_acid_code{$first_aa};
			my $second_num = $amino_acid_code{$second_aa};
				if ($first_num < $second_num){
					$score = $blosum_array[$second_num][$first_num];
				}
				if ($first_num == $second_num){
					$score = $blosum_array[$second_num][$first_num];
				}
				if ($second_num < $first_num){
					$score = $blosum_array[$first_num][$second_num];
				}
				if (($j != $k) && ($score >= 0) && ($first_num != 21) && ($second_num != 21)
&&
					($first_num != 20) && ($second_num !=20)){
					$similarity = $similarity + 1;
				}
				if (($j != $k) && ($first_num == $second_num) &&  ($first_num != 21) &&
($second_num != 21)
					&& ($first_num != 20) && ($second_num != 20)) {
					$identity = $identity +1;
				}
				if (($j == 0) && ($j == $k)){
					if ($first_num == 21) {
						$seqq1 = $seqq1 - 1;
					}
				}
				if (($j == 0) && ($j < $k)){
					if ($second_num == 21) {
						$seqq1 = $seqq1 - 1;
					}
				}
			$final_score = $final_score + $score;
		}
		if ($similarity > 0){
			$similarity_a = ($similarity + 1) * 100 / $seqq1;
		}
		if ($identity > 0){
			$identity1 = ($identity + 1) * 100 / $seqq1;
		}
		$similarity_aa = $similarity_aa + $similarity_a;
		$similarity = 0;
		$similarity_a = 0;
		$identity2 = $identity2 + $identity1;
		$identity = 0;
		$identity1 = 0;
```

```
        }
        ##                                                                                                ##
        ##################################################################################################

        if  ($seqq1 != 0){
                $similarity_aa = $similarity_aa / $seqq1;
                $identity_counter = $identity2 / $seqq1;
        }
        if ($seqq1 == 0) {
                $similarity_aa = 100;
                $identity_counter = 100;
        }

        push (@score_array, $final_score);
        push (@similarity_array, $similarity_aa);
        push (@identity_array, $identity_counter);
}
##                                                                                                ##
####################################################################################################

####################################################################################################
## Calcultaion of total blosum score, similarity and identity in the sliding window          ##

my @temp_score_array = ();
for (my $m =0; $m < $N; $m++){
        my $temp_score = shift @score_array;
        push (@temp_score_array, $temp_score);
}

my $m = 0;
my $final_loop_counter = $counter - $N +1;
for (my $p = 0; $p < $final_loop_counter; $p++){
        my $window_score = 0;
        foreach my $score (@temp_score_array){
                $window_score = $window_score + $score / $N;
        }
        print $window_score."\n";
        shift @temp_score_array;
        push (@temp_score_array, shift @score_array);
}

print "\n\n\n\n";
my $m = 0;
my @temp_similarity_array = ();
for (my $m =0; $m < $N; $m++){
        my $temp_similarity = shift @similarity_array;
        push (@temp_similarity_array, $temp_similarity);
}
```

```
my $p = 0;
my $final_loop_counter = $counter - $N +1;
for (my $p = 0; $p < $final_loop_counter; $p++){
        my $window_similarity = 0;
        foreach my $similarity_aa (@temp_similarity_array){
                $window_similarity = $window_similarity + $similarity_aa / $N;
        }
        print $window_similarity."\n";
        shift @temp_similarity_array;
        push (@temp_similarity_array, shift @similarity_array);
}

print "\n\n\n\n";
my $m = 0;
my @temp_identity_array = ();
for (my $m =0; $m < $N; $m++){
        my $temp_identity = shift @identity_array;
        push (@temp_identity_array, $temp_identity);
}


my $p = 0;
my $final_loop_counter = $counter - $N +1;
for (my $p = 0; $p < $final_loop_counter; $p++){
        my $window_identity = 0;
        foreach my $identity (@temp_identity_array){
                $window_identity = $window_identity + $identity / $N;
        }
        print $window_identity."\n";
        shift @temp_identity_array;
        push (@temp_identity_array, shift @identity_array);
}

##                                                                                            ##
################################################################################################
```

BLOSUM 62 Matrix (1) was modified as described in the paper

	A	R	N	D	C	Q	E	G	H	I	L	K	M	F	P	S	T	W	Y	V	-	X
A	4																					
R	-1	5																				
N	-2	0	6																			
D	-2	-2	1	6																		
C	0	-3	-3	-3	9																	
Q	-1	1	0	0	-3	5																
E	-1	0	0	2	-4	2	5															
G	0	-2	0	-1	-3	-2	-2	6														
H	-2	0	1	-1	-3	0	0	-2	8													
I	-1	-3	-3	-3	-1	-3	-3	-4	-3	4												
L	-1	-2	-3	-4	-1	-2	-3	-4	-3	2	4											
K	-1	2	0	-1	-3	1	1	-2	-1	-3	-2	5										
M	-1	-1	-2	-3	-1	0	-2	-3	-2	1	2	-1	5									
F	-2	-3	-3	-3	-2	-3	-3	-3	-1	0	0	-3	0	6								
P	-1	-2	-2	-1	-3	-1	-1	-2	-2	-3	-3	-1	-2	-4	7							
S	1	-1	1	0	-1	0	0	0	-1	-2	-2	0	-1	-2	-1	4						
T	0	-1	0	-1	-1	-1	-1	-2	-2	-1	-1	-1	-1	-2	-1	1	5					
W	-3	-3	-4	-4	-2	-2	-3	-2	-2	-3	-2	-3	-1	1	-4	-3	-2	11				
Y	-2	-2	-2	-3	-2	-1	-2	-3	2	-1	-1	-2	-1	3	-3	-2	-2	2	7			
V	0	-3	-3	-3	-1	-2	-2	-3	-3	3	1	-2	1	-1	-2	-2	0	-3	-1	4		
-	-5	-5	-5	-5	-5	-5	-5	-5	-5	-5	-5	-5	-5	-5	-5	-5	-5	-5	-5	-5	0	
X	0	0	0	0	0	0	0	0	0	0	0	0	0	0	0	0	0	0	0	0	0	0

NOTE:
- indicates a loop
X indicates a missing amino acid

(1)Dayhoff, M.O., Schwartz, R.M. & Orcutt, B.C. (1978) "A model of evolutionary change in proteins."
In "Atlas of Protein Sequence and Structure, vol. 5, suppl. 3."
M.O. Dayhoff (ed.), pp. 345-352, Natl. Biomed. Res. Found., Washington, DC.
